# A causal inference and Bayesian optimisation framework for modelling multi-trait relationships—Proof-of-concept using *Brassica napus* seed yield under controlled conditions

**DOI:** 10.1371/journal.pone.0290429

**Published:** 2023-09-01

**Authors:** Alexander Calderwood, Laura Siles, Peter J. Eastmond, Smita Kurup, Richard J. Morris

**Affiliations:** 1 Department of Computational and Systems Biology, John Innes Centre, Norwich, Norfolk, United Kingdom; 2 Plant Sciences and the Bioeconomy, Rothamsted Research, Harpenden, Hertfordshire, United Kingdom; Government College University Faisalabad, PAKISTAN

## Abstract

The improvement of crop yield is a major breeding target and there is a long history of research that has focussed on unravelling the mechanisms and processes that contribute to yield. Quantitative prediction of the interplay between morphological traits, and the effects of these trait-trait relationships on seed production remains, however, a challenge. Consequently, the extent to which crop varieties optimise their morphology for a given environment is largely unknown. This work presents a new combination of existing methodologies by framing crop breeding as an optimisation problem and evaluates the extent to which existing varieties exhibit optimal morphologies under the test conditions. In this proof-of-concept study using spring and winter oilseed rape plants grown under greenhouse conditions, we employ causal inference to model the hierarchically structured effects of 27 morphological yield traits on each other. We perform Bayesian optimisation of seed yield, to identify and quantify the morphologies of ideotype plants, which are expected to be higher yielding than the varieties in the studied panels. Under the tested growth conditions, we find that existing spring varieties occupy the optimal regions of trait-space, but that potentially high yielding strategies are unexplored in extant winter varieties. The same approach can be used to evaluate trait (morphology) space for any environment.

## Introduction

Crop plants are often studied from the perspective of how to increase yield. Yield is known to be a complex trait, depending on multiple genes, plant architecture and plant-environment interactions. Total seed yield can be decomposed into multiple simpler yield components or yield traits [1]. Based on these yield components, plants can be considered as points in a trait-space in which each dimension is a considered trait. Within this representation, determining how different traits affect yield corresponds to identifying a function over the domain of trait-space that maps a point (yield traits) to a number (yield). These yield traits often exhibit complicated trait-trait relationships due to underlying physiological interactions [2–6]. Such dependencies between the traits mean that the full trait space is not accessible. Instead, plants (as a combination of traits) exist on a manifold within trait space, which is defined by the constraints imposed by causal relationships between traits. These causal relationships are a function of the environment and due to its dynamic nature, any yield manifold must be viewed as non-stationary.

**Fig 1:**
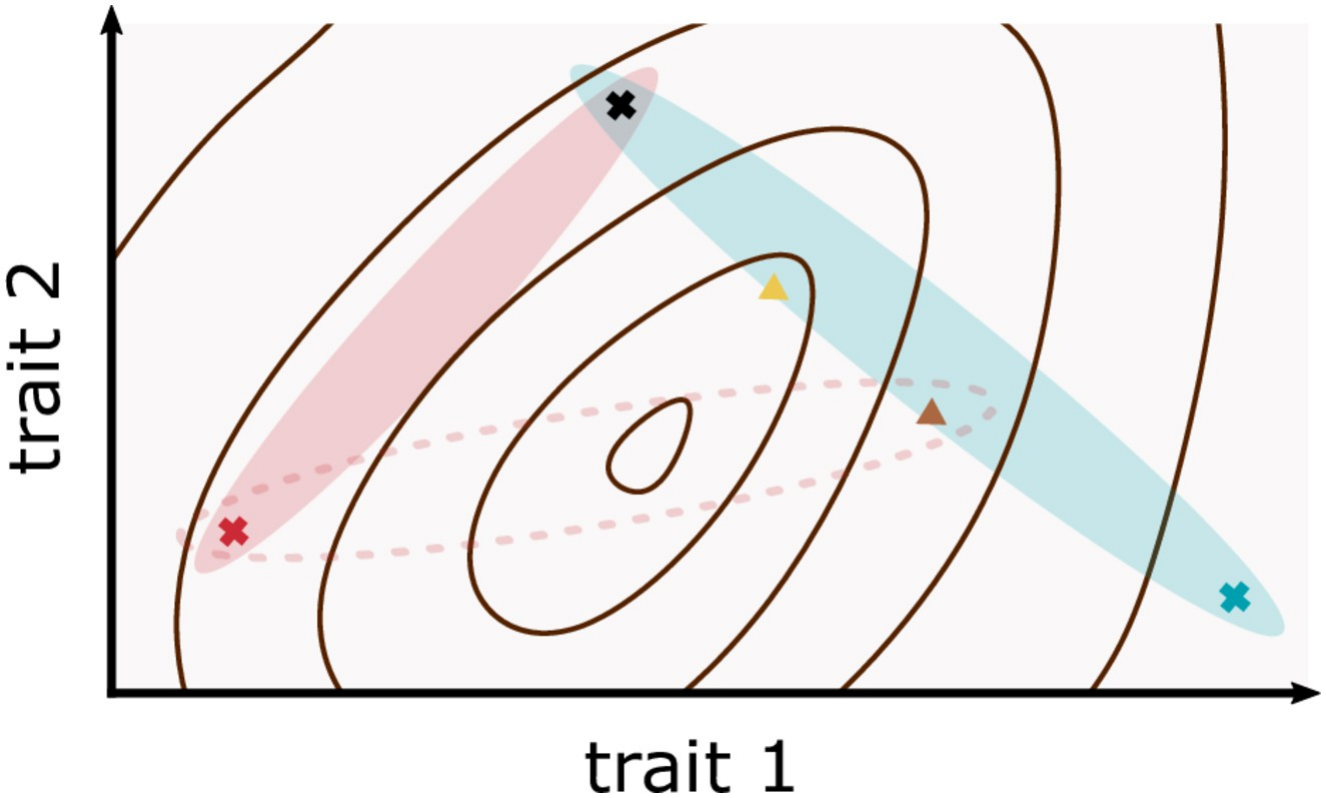
Identification of the yield surface facilitates selection of parent varieties for crosses, and selection among the progeny of a cross. A cartoon showing trait-space, in which yield is a function of trait 1 and trait 2, indicated by contours with a maximum value in the middle of the plot. Plant varieties occupy points in the trait-yield space, marked as coloured crosses or triangles. Candidate parental lines are shown as crosses, offspring lines are shown as triangles. The trait values of potential offspring of a particular parental cross are abstracted as ovals in trait-space, reflecting that progeny phenotypes are not a linear combination of parent values, but are constrained by them. Even in this simple convex yield function example, greedy selection can be inefficient. In deciding between crossing either the red or blue candidate parent line with black parent, greedy selection favours crossing the red, as this yields higher than the blue parent. However, the yield surface shows that progeny from a blue-black cross can be expected to include higher yielding varieties than from a red-black cross, as the blue oval covers a region of trait space with a higher maximum yield than the red oval. Knowledge of the yield surface is also useful in selecting which progeny of a cross to take forward. The yellow triangle plant is the highest yielding among progeny of the blue-black cross, but the suboptimal brown plant is worth retaining, as it can be crossed with the red plant to produce the best plants of all.

Crop improvement through genetic selection corresponds to the choice of traits with the highest yield. This process can be viewed as a search for the point on the manifold which results in the highest yield potential. To achieve this, trait space is sampled by iterating the two steps of 1) making crosses between chosen parent varieties, and then 2) selecting within the progeny based on desired attributes for further evaluation [7]. These steps are expensive, motivating the need for an efficient exploration method (providing maximum information from a given number of samples). Two fundamentally different approaches to the search problem are possible [8]. In the first approach, crosses and selection are made using existing plants which perform better than their rivals. Performance may be evaluated directly, as the magnitude of a trait of interest (predominantly some measure of total yield), or indirectly from other traits which are considered desirable due to their association with total yield [1, 9–13]. This approach is essentially a so-called greedy algorithm, in which a locally optimal choice is made at each iteration. A combination of locally optimal choices may, however, fail to find the globally optimal solution [14]. Crossing high-yielding parents without understanding why they are high-yielding could, therefore, lead to the eventual perfection of a suboptimal yield strategy. Even if the yield potential surface is convex, a greedy crossing strategy is potentially inefficient as crossing the highest-yielding parents, may lead to a suboptimal next generation (Fig 1). The second approach is to define (from biological expertise based on a physiological understanding) the yield trait values of the ideal hypothetical plant which is expected to produce maximum yield (an ideotype, [15]), and which is then bred towards. Given the knowledge of how yield varies as a function of other traits, the ideotype strategy has the potential to outperform the greedy approach. Suboptimal parent varieties with complementary traits can be selected to produce the optimal next generation, at the cost of measuring multiple yield component traits. However, a challenge with this strategy is the determination of an ideotype as, based on biological expertise alone, the complexity of the causal relationships between traits makes ideotype predictions extremely difficult. Furthermore, it is worth noting that given the changing nature of the environment, trait manifolds and ideotypes are dynamics entities, although we will approximate them as static within the current development.

Causal relationships due to physiological linkage between traits within an individual plant have been experimentally demonstrated through perturbation experiments [2–6, 16–18]. These relationships between traits may be based on developmental processes (e.g. number of seeds is dependent on the number of ovules because one develops into the other), resource limitation (e.g. pod number, seed number per pod, and seed size are not independent, [1]), or intra-plant signalling (e.g. seed derived signalling cascades trigger localised pod expansion [19, 20]. This definition does not include correlative relationships due to genetic linkage or pleiotropic gene action. It is not clear that these physiological relationships can be overcome and must therefore be accounted for in plant breeding strategies.

Complex, non-linear trait-trait interactions mean that i) a particular trait value may be beneficial for yield in combination with one set of traits, but detrimental given another [21], ii) that traits may have an optimal value rather than being generally beneficial or detrimental (i.e. that they have a non-monotonic relationship with yield), and iii) that changes in one trait may cause changes in other traits which partially or completely nullify the anticipated effect on yield [2–6, 16, 17]. Based on biological intuition alone, it is therefore challenging to assess whether increasing or decreasing the value of a trait can be expected to increase total yield. Mathematical modelling can help define an ideotype by i) identifying the plausible trait-trait relationship structure given current data, ii) identify the feasible region of trait-space that plants can occupy, iii) predicting the sensitivity of yield to trait modification and the optimal set of traits to maximise yield and iv) proposing the most informative new data to collect to further refine the model [22–27].

When a theoretical ideotype is identified in terms of a single desirable trait, then the heritability of the target trait must be confirmed to ensure it can be produced through selective breeding. When an ideotype is defined in terms of multiple traits, the independent heritability of these traits may be important to ensure that the ideotype can be produced through selective breeding. Sources of variation in a trait can be partitioned into genetic, environmental and stochastic variability [28]. However, when causal trait-trait relationships are considered, sources of variation can alternatively be partitioned into the “direct” effects (of genetics, environment, and inherent variability) on the trait of interest, and the “indirect” effects that these factors have on the trait of interest via their influence on other traits which cause it. By modelling the relationships between traits, “direct heritability” (the heritability of the trait, conditioned upon other traits under selection) and “indirect heritability” (heritability caused by the heritability of the traits which affect it) can be estimated.

Genome Wide Association Studies (GWAS) are used to identify candidate alleles with genetic variation underlying yield traits. Trait-trait relationships also have complicating effects on these studies, and it has previously been shown that crop models can be used to constrain genomic prediction models to improve their performance [29, 30]. Mediated pleiotropy occurs when phenotypes are causally related, and so genetic loci associated with the first (causal) phenotype are also statistically associated with the second [18, 19, 31–33]. Distinguishing biological pleiotropy from mediated pleiotropy is valuable to avoid the misleading identification of genes as being directly involved in a trait of interest but which, in fact, act indirectly via a different trait. Biological pleiotropy can be distinguished from mediated pleiotropy by controlling for the traits that cause the traits of interest before testing for genetic association. For example by performing GWAS on the residuals of a trait-trait relationship model [32, 34].

Here, we explore the consequences of causal trait-trait relationships for crop improvement by modelling their effect on predictions for yield improvement, trait heritability and genetic association studies. Oilseed rape is an excellent system for studying trait-trait relationships due to its complicated and plastic developmental morphology [1–3, 35], and the availability of diversity panels. It is also not clear that yield in oilseed rape has yet been optimised to the extent of (for example) wheat. We apply sequential path analysis [25] to macro-traits (measured at the whole plant level) and micro-traits (comprising ovule area and number, gynoecia, ovary and style length) of individual, pot-grown plants to identify potential relationships between previously characterised morphological traits, as well as the less studied female reproductive traits [12, 16, 36–39]. We model the trait-yield function to identify which traits are expected to alter total seed weight in spring and winter oilseed rape plants, whilst accounting for compensatory or exacerbating changes in other traits. We then apply Bayesian optimisation over the modelled relationship between seed yield and plant morphology to propose the optimal next generation of plants in terms of multiple yield traits. These correspond to promising high-yield oilseed rape ideotypes (under the glasshouse growth conditions that were used to generate the data), in regions of trait-space which are underexplored in the studied panel of plants. Within this proof-of-concept study, we also find that by conditioning on physiologically causal traits, we can identify which traits are independently heritable, and increase the power to detect genetic variants associated with a trait of interest. Such inferences are conditional on the data and in particular on the environmental conditions. Our study should be interpreted in this light, and more data collected under realistic conditions is required to make useful inferences about crop performance in the field.

## Methods

### Plant growth conditions and trait measurement

The studied *B*. *napus* diversity set consisted of 94 varieties [40, 41]. The population was classified in 4 oilseed rape groups, including Winter oilseed rape (41 varieties), Spring oilseed rape (22 varieties), Semi-winter oilseed rape (8 varieties) and Others (23 varieties) which included swede, Siberian kale, unspecified and fodder varieties, (S1 File). Spring and Winter oilseed rape groups were used to fit separate trait-trait models, as different relationships between trait and yield exist in these groups [1, 42]. For GWAS analysis, a single model for all available varieties was used to maximise the power to detect SNP-trait associations.

Plants were pot-grown as described in [42] arranged in 2 glasshouses. Each glasshouse contained all 94 varieties arranged in a 20x12 non-resolvable row-column design. All varieties were replicated either 2 or 3 times per glasshouse to give a total of 5 replicates across both glasshouses. The design was generated in CycDesigN (CycDesigN 6.0, VSN International Ltd, Hertfordshire, UK). A total of 27 traits were measured with either 3 or 5 biological replicates for microtraits and macrotraits, respectively (S1 Table). Phenotyping of these traits was as described in [42].

### Data pre-processing

Transformations were applied to each trait to make them more normally distributed, S2 Table. Missing values were imputed by predictive mean matching, using the “mice” (v3.11.0) R package [43] as detailed in S1 File “impute_data.R”.

### Identification of trait relationship structure

**Fig 2:**
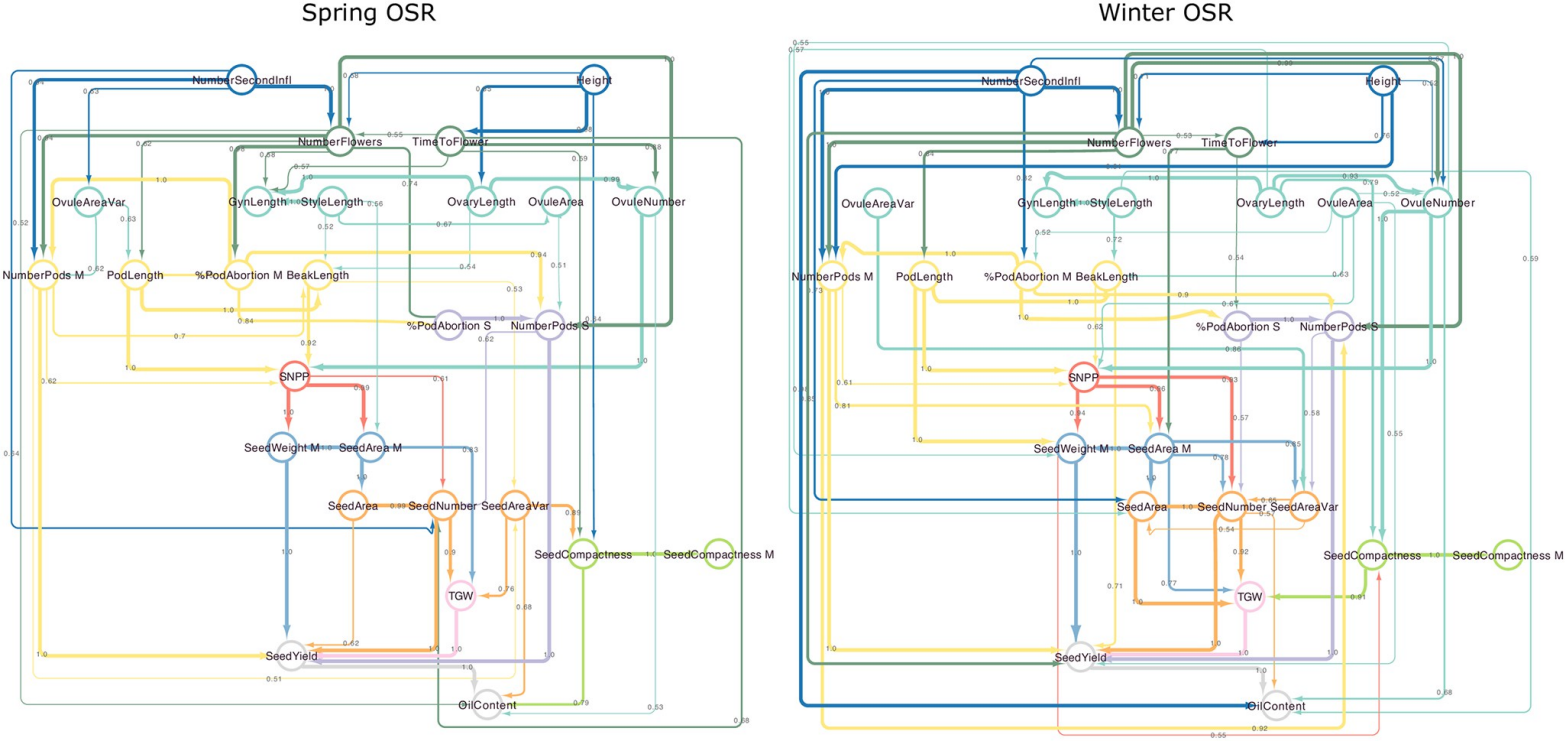
Graphical models for inferred relationships between traits in spring and winter oilseed rape. Observed traits are shown as nodes, edges link traits which are inferred to be directly associated, without mediation by other observed traits. Edge directionality is from the inferred causal trait (parent trait) to the caused trait (child trait). Node position and colour corresponds to the predefined hierarchy of traits (see Methods). Edge colour corresponds to the source node colour. Edge width and annotation show the estimated probability of the existence of that trait-trait relationship.

To learn the structure of the relationships between the measured traits, Sequential Path Analysis models [24] were separately fit for spring and winter oilseed rape varieties. Based on previous biological knowledge of oilseed rape development, permitted trait-trait relationships were defined, and a small set of known trait-trait links identified, which must be included in any learnt model structure (see S2 File). To learn the trait relationship structure within these constraints, a Gaussian Bayesian Network was fitted using the bnlearn R package [44], such that each node is the dependent variable in an additive linear model in which its parent nodes are the independent variables. The possible network structure space was explored by Tabu search, seeking to maximise the Bayesian Information Criterion score of the trait-trait model. Bootstrap sampling of the data (number of replicates = 500) was used to empirically estimate the probability of each inferred trait-trait link based on the frequency of its identification in the best model for each sample. To reduce the risk of overfitting, the data was randomly split into five folds, and the above modelling process carried out separately on each fold. The inferred models were averaged (following [45]) to only include links identified consistently across folds (inferred model structures shown in Fig 2).

### Predicting the consequence of modifying traits on seed yield

The inferred trait relationship structures for spring and winter oilseed rape (directed acyclic graphs, DAGs, Fig 2) was used to define the independent variables in non-linear regression models for each observed trait. Here, we use a familial convention to describe relationships between nodes, such that in the graph A -> B -> C, A is the parent of B, which is B’s child. Nodes which can be reached by following the directed links starting at a node of interest are referred to as its descendants, so B and C are both descendants of A.

Each trait (node in the DAG) was considered as a dependent variable (***f***), with its parent nodes the corresponding independent variables (***X***). Their relationship was modelled using Gaussian Process (GP) regression,

$$P\left(f\;|\;X\right)=N\text{(}f\;|\;\mu ,\;K),$$

where *N* denotes a normal distribution over ***f*** with mean ***μ*** and covariance ***K***. The covariance matrix ***K*** was defined using the Automatic Relevance Determination kernel,

$$K_{ij}=\alpha^{2}\text{exp}(-\frac{1}{2}\overset{q}{\underset{k=1}{\sum }}{\left(\frac{x_{ik}-x_{jk}}{\rho_{k}}\right)}^{2})+\delta_{ij}\sigma_{i}^{2}$$

where *q* is the number of parent trait nodes (independent variables), and *δ*_*ij*_ is Kronecker’s delta (1 if *i* = *j* and 0 otherwise). GP hyperparameters *α*, *ρ*, *σ* were estimated through regularised maximum marginal likelihood, with priors *α* ∼ *N*(0,1), *ρ*_*k*_ ∼ *IG*(5,5), *σ*_*i*_ ∼ *N*(0,1). To estimate posterior probabilities of dependent variables, No-U-Turn sampling was carried out, implemented in Stan [46] using 4 chains, with 1000 iterations burn-in and 1000 sampling iterations.

To predict the direct and indirect effects of individually modifying each trait on downstream traits, the trait of interest was sequentially fixed to modified values between the experimentally observed extreme values, and the estimated posterior values of its direct children in the DAG (given the modified value) sampled. These sampled predictions were then used as inputs to the next level of GP models, (with response and explanatory variables as defined by the DAG structure), to estimate the values of the modified-trait’s grandchildren and so on, respecting the dependencies identified in the DAG, until all descendants of the modified node were predicted. This sampling approach was used to allow uncertainty in predictions to correctly propagate through the DAG structure. Traits which were not descendants of the modified trait were held to their observed *in planta* values. The predicted effect of modifying each trait in spring and winter oilseed rape were averaged over the five candidate trait-relationship DAG structures identified type.

### Bayesian optimisation for ideotype identification

To identify optimal crop ideotypes for maximum seed yield and propose other promising regions of trait space for exploration, we used a Bayesian Optimisation framework. As the “surrogate model” we used sub-models of the GP models for predicted seed yield described above, such that seed yield was predicted only from its parent traits (shown in Fig 2).

The relationship between seed yield and these parent traits was modelled by GP regression as before. As the “acquisition function” for maximisation we used Expected Improvement (EI) [47] defined as

$$EI\left(x\right)=E\left[\text{max}\left(f\left(x\right)-f(x^{+}\right),\;0)\right],$$

where *f*(*x*) is the predicted value of seed yield at *x*, and *x*^+^ is the observed point in trait-space with the maximum seed yield. Expected Improvement thus allows us to balance exploitation of regions with high predicted seed yield, with exploration of regions with high posterior uncertainty. Proposed trait values were constrained to be between the maximum and minimum observed values. Because generating crosses is costly, but parallelizable, it is desirable to be able to propose multiple optimal points for exploration given the current observed data. In order to obtain an approximately optimal design for *q* proposed sampling points, we used the “Constant Liar” heuristic strategy proposed in [48]. Briefly, proposed sampling points were generated sequentially, such that when generating each new point, previously proposed, (but still untested) sampling points were assumed to have value L, here L was set as the maximum experimentally observed seed yield in Spring or Winter OSR.

Relationships between the traits exist in the observed data, and so it is unlikely that all points of trait-space can be occupied by a real plant. To incorporate correlation between the parent traits as constraints in the optimisation of proposed points, we followed the approach proposed in [49]. An independent (correlation free) basis for the trait-space was identified by PCA of the parent traits of seed yield. PCs sufficient to explain >95% of variation were used as predictors, and Bayesian optimisation was carried out using this new independent basis as described above. Predictions in the PC basis were then transformed back to the observed trait basis for reporting.

### Genome-wide association analysis

Publicly available RNA-seq derived SNPs [41], were filtered for Minor Allele Frequency > 0.05 among the 94 varieties which form the intersection of the varieties for which trait data and RNA-seq data was collected. This resulted in 108,653 SNPs which were found to correspond to oilseed rape groups (S1 Fig). These were used for GWAS with the directly observed traits, and the trait-trait model residuals. Model residuals were calculated from a single trait-trait model inferred from all 94 varieties as described above for spring and winter OSR.

STRUCTURE [50], and Structure Harvester [51] were used to calculate population structure (Q). TASSEL 5.0 [52] was used to calculate first 5 or 10 genotype principle components (PCA5d, PCA10d), and kinship matrix (K). Multiple models and methods of accounting for population structure were tested for their control false positive and false negative associations in the data. TASSEL 5.0 was used to fit GLM-Q, GLM-PCA5d, GLM-PCA10d general linear models (GLM), and MLM-K, MLM-Q+K, MLM-PCA5d+K mixed linear models (MLM). A Bayesian-information and Linkage-disequilibrium Iteratively Nested Keyway (BLINK) model was fit using GAPIT [53, 54]. QQ-plots were used to select the most appropriate model (S2 Fig). Based on these, GLM-PCA5d was used for NumberPods M and OilContent.

The significance of associations between SNPs and traits was based on the threshold p < 5.8x10^-6^, which results from a false discovery rate of 0.1, correcting for multiple testing following [55] with a parameter a D’ of 0.7. A previously published homolog mapping between Brassica and Arabidopsis genes was used [41].

### Consequences of trait relationships for heritability and genetic variant association

See Supplemental Methods (S3 File) for details of the regression analyses used to ascertain to consequences of identified trait-trait relationships on estimated heritability, and details of the simulated GWAS analysis.

### Code availability

All code used in data processing, modelling and analysis is made available at https://github.com/AlexCalderwood/identification-of-ideotypes, together with exemplar input and output data files.

## Results

### Inference of trait-trait relationship structure

**Fig 3:**
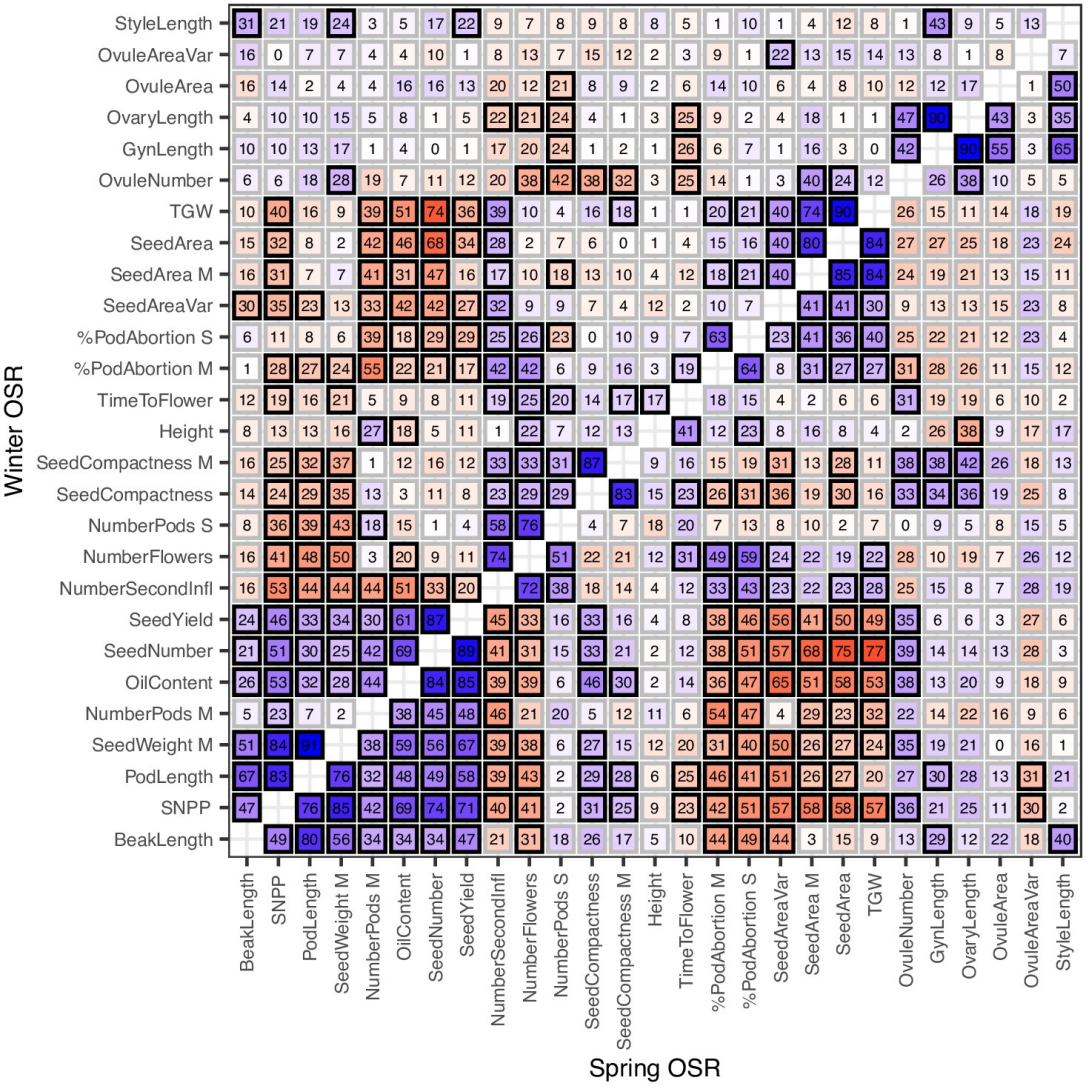
Correlation between traits in spring and winter oilseed rape varieties. Spearman’s correlation coefficients between traits for Winter (above diagonal) and Spring (below diagonal) oilseed rape varieties. Blue shows positive correlation, red shows negative. Reported values are absolute values of coefficients, multiplied by 100. A black border indicates statistically significant, non-zero correlation (t-test), using Benjamini-Hochberg adjusted p-value with significance level 0.05.

Oilseed rape exhibits complex morphology with many traits which might be expected to affect seed yield. We measured 27 traits in spring and winter oilseed rape to examine the relationships between them and with seed yield. We modelled spring and winter oilseed rape varieties separately, as studies conducted in spring type and winter type oilseed rape have reached different conclusions regarding which features are key for crop yield [1, 37, 42], and we observed differences in trait-trait correlations (Fig 3) between these groups.

From the correlation matrix it can be seen that many traits are statistically dependent (Fig 3), yet the causal structure underlying the relationships between the traits is not obvious. To identify the structure of the trait-trait relationship graph, we applied sequential path analysis [25], which explains these correlation relationships in the most parsimonious way (see Methods). Fig 2 shows the DAG structure of the inferred model relating traits and seed yield (SeedYield) in spring and winter oilseed rape. Traits which are connected directly were inferred as having a direct causal relationship, not mediated by the other observed traits. Nodes which are indirectly linked have an indirect relationship, which are mediated by the traits (nodes) between them. For example, in spring oilseed rape, the number of flowers (NumberFlowers) directly affects the number of pods (NumberPods S, NumberPods M), but also affects seed yield indirectly, via the number of pods. The inferred graphs are consistent with well-known relationships, for example in both spring and winter oilseed rape, the number of flowers (NumberFlowers) is affected by the number of secondary inflorescences (NumberSecondInfl) [56] and the number of pods on the main, and secondary branches (NumberPods M and NumberPods S) are consequences of the number of flowers and pod abortion (%PodAbortion) [57].

### Association of individual traits with yield

**Fig 4:**
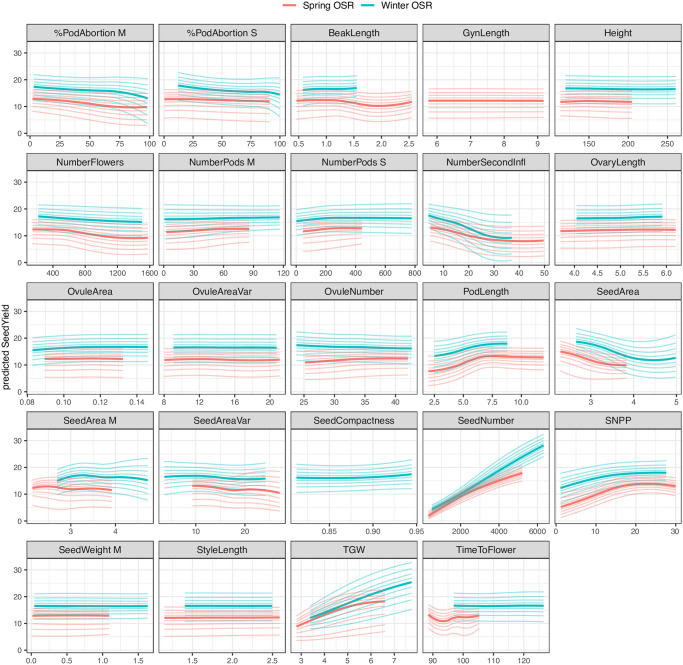
For many yield traits there is insufficient evidence to conclude that their individual modification will generally alter yield. Trait-trait models were used to predict the effect of altering yield trait values in Spring and Winter oilseed rape assuming directly linked traits are linked by causal relationships. Each facet shows the predicted effect of changing one yield trait on seed yield between the minimum and maximum values observed for that trait. Traits downstream of the altered trait in the model DAGs were predicted, and so allowed to vary in consequence of the changes. All traits upstream of (or disconnected from) the varied trait were held to their observed values. Only plots for traits which are upstream of seed yield in the modelled DAGs (and which therefore may affect it) are shown. Yield values were predicted for all varieties in each panel. Median predicted seed yield values are shown in heavy line. Predicted seed yield quantiles between 10% and 90% at 10% intervals are plotted with light lines. Uncertainty is a consequence of both uncertainty in the modelled relationships, and variation between the varieties in each panel. Spring oilseed rape is shown in red, winter oilseed rape in blue.

We used the inferred graph structures to model the effect that changing yield traits can be expected to have on seed yield in spring and winter oilseed rape, accounting for non-linear, and interactive effects (see Methods). Fig 4 shows predicted seed yield for spring and winter oilseed rape as each trait is varied individually, whilst holding upstream traits to their observed values, allowing downstream traits, and seed yield to vary due to the altered trait.

**Fig 5:**
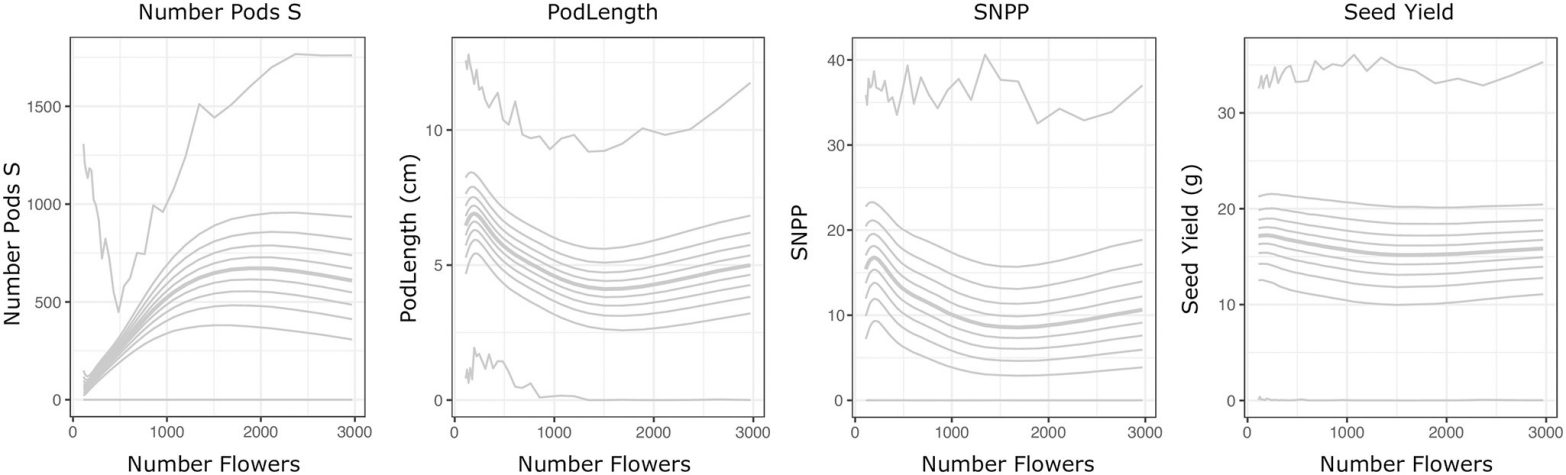
Compensatory interactions between yield traits buffer the effects of many yield traits. The predicted effects in Winter oilseed rape of modifying the number of flowers on the number of pods on secondary branches (Number Pods S), average pod length (PodLength), seed number per pod on the main branch (SNPP), and seed yield per plant (Seed Yield). Median value and 10% quantiles in the response trait predictions are shown. Although increasing the number of flowers is expected to result in the production of more pods (up to a point), compensatory changes in pod length and seed number per pod result in an expected slight reduction in final seed yield.

Surprisingly, we see little clear evidence that individually modifying many previously identified yield traits will affect seed yield. The traits which are not predicted to affect Seed Yield can be divided into several classes based on whether they correlate with seed yield (Fig 3), and whether a causal path exists between them and seed yield in the model structure (Fig 2). Many traits are highly correlated with yield, but their modification is in fact not expected to affect it. Instead, these traits have a common causal factor with yield. For example, in winter oilseed rape the correlation between beak length and seed yield is due to pod length, which is modelled as causing both yield and beak length. Consequently, modifying beak length directly is not expected to affect seed yield, whereas modifying pod length is expected to affect several traits, including beak length and seed yield, (S3 Fig). In another group are traits that are not predicted to affect seed yield, although causal paths do exist between them in the model DAG structure. For example, the number of flowers (NumberFlowers) in winter oilseed rape. Members of this group are predicted to not affect yield due to compensatory changes in other traits. For example, although a greater number of flowers is predicted to result in a greater number of pods, the model correctly identifies the well-known negative relationship between pod number and size [58]. These numerous pods are therefore expected to be shorter, and each contain fewer seeds, resulting in little overall predicted effect on seed yield (Fig 5).

The traits whose alteration are most confidently predicted to modify seed yield are the number of secondary inflorescences (NumberSecondInfl), pod length (PodLength), total seed number (SeedNumber), seeds per pod (SNPP), thousand grain weight (TGW) and seed area (Fig 4). Interestingly, increasing total seed number (SeedNumber) is expected to lead to increased seed yield, despite identification of the well-known negative relationship between seed number and TGW (S3 Fig, [4]). Note that although increasing TGW is also expected to increase seed yield, this is under the assumption that seed number (as an upstream node of TGW in the causal network) is held constant and so does not take account of the relationship between them.

Fig 4 shows several examples of non-linear relationships between seed yield and yield traits, for example the number of secondary inflorescences, seed area, and seed number per pod, all have non-linear relationships with predicted seed yield. It is therefore not clear that generalised recommendations based on linear models that a particular trait should be selected for or against can be applied across varieties, but instead should account for the existing value of those traits in the varieties undergoing improvement. The exceptions to this are total seed number (SeedNumber) and thousand grain weight (TGW), which do not exhibit saturation behaviour in the expected yield response within the observed trait range (at least in winter oilseed rape).

### Identification of optimal multivariate oilseed rape ideotypes accounting for trait-trait relationships

Having identified the traits expected to affect seed yield when varied individually, we were interested to identify crop ideotypes in terms of multiple traits simultaneously accounting for interactions between them. To identify ideotype plants for our experimental conditions, we performed Bayesian optimisation over trait values to maximise the expected improvement (EI) in seed yield (see Methods). The EI metric identifies points which (when their predicted yield distribution is integrated) have the greatest probability of achieving higher seed yield than the maximum seed yield among the observed plants. It therefore balances the search of high yielding regions of trait space, with exploration of regions of greater uncertainty in the predicted yield distribution. To reduce the dimensionality of the trait space optimised over, only traits which directly affect seed yield (traits which connect to it directly in Fig 2) were optimised. Although, modification of indirectly acting traits is also expected to affect seed yield (Fig 4), their action is only via directly connected traits. Consequently, if the traits directly connected to seed yield are controlled, then all other traits are irrelevant to seed yield.

If the empirically observed relationships between the yield traits are ignored, then unsurprisingly, optimisation finds many ideotype plants that are expected to have higher seed yield than the experimentally observed plants in both the spring and winter oilseed rape panels (S4 Fig). Predominantly the proposed ideotypes produce very large numbers of very, or moderately heavy seeds, and a broad range of values for the other yield traits. However, this relaxation of the observed correlations among traits is unlikely to be reasonable as previous experimental work indicates that they are due to intra-plant competition for seed filling resources [2–6, 16, 17].

**Fig 6:**
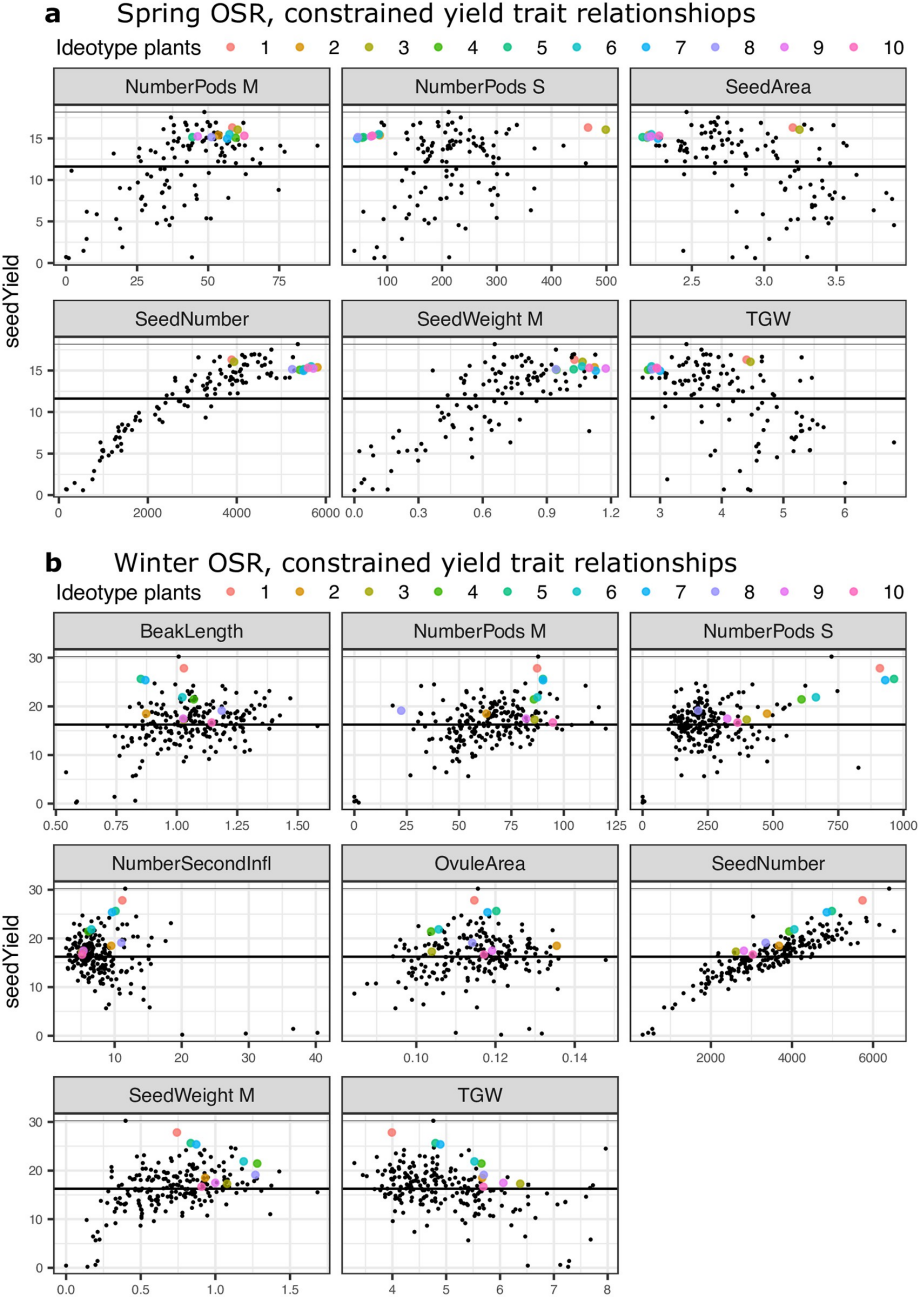
Model identified optimal crop ideotypes. Facets show traits which affect seed yield directly in the **a)** Spring and **b)** Winter oilseed rape panels. Coloured points show mean predicted seed yield for identified hypothetical plants with the indicated yield trait values. Their order reflects their probability of having greater seed yield than the best experimentally observed plant, with 1 having the best chance, and 10 the worst of the calculated ideotypes. These points are identified through Bayesian optimisation as the unobserved points with the best chance of exceeding the maximum observed seed yield in each panel. Black points show seed yield and yield trait values for experimentally observed plants. The thin black line marks the maximum seed yield observed amongst the observed plants, the thick black line shows mean seed yield among the observed plants.

Fig 6 shows the observed yield of experimental plants, and the expected seed yields of ideotype plants when empirically observed relationships between yield traits are respected as additional constraints during the optimisation of EI (see Methods). In spring oilseed rape, many observed plants are higher yielding than the expected values of any hypothetical plants. This indicates that when yield trait relationships are constrained to follow their observed relationships, the optimal regions of trait space under these growing conditions are already well explored by existing plants. The high-yield region is broadly defined by the production of a large number of seeds (SeedNumber > 3000), and a large proportion of productivity via the primary inflorescence relative to the spring oilseed rape plants in the panel. Seeds produced on the main inflorescence are relatively heavy (SeedWeight M > 0.6g), but the weight of seeds produced over all inflorescences (TGW) of high performing plants does not appear to be tightly constrained and covers most of the range observed among all spring oilseed rape plants. The number of pods on the main inflorescence is slightly above the average for the panel, ranging from approximately 35 to 60. The number of pods on other branches (NumberPods S), and seed area (SeedArea) are more variable, but slightly lower than the average in this spring oilseed rape population. Hence, a focus on production via the main inflorescence appears to be required for good performance.

Conversely, among winter oilseed rape, only one observed plant yields higher than the mean expected seed yield of the best hypothetical point. This suggests that the optimal yield trait region under these growth conditions may not have been fully explored by varieties in the winter oilseed rape panel. In winter oilseed rape increased seed number is expected to lead to increased seed yield, and the best ideotypes produce the most total seeds (seedNumber) of small size (TGW). Ideotype production on the main inflorescence is suggested to be similar to that observed among the experimentally high yielding plants. The best ideotypes have approximately 90 pods on the main inflorescence and produce 0.75 to 0.9g of seed per 10 pods on the main stem (seedWeight M). Whilst the identified ideotypes respect the trade-off between seed number and TGW, the most promising ideotype plants occupy the upper edge of the cloud of experimentally observed plants in both these metrics. This means that they are expected to be able to produce heavier seeds than observed plants which produce the same number of seeds, and more seeds than plants which produce the same TGW. They have high values for traits associated with increased photosynthetic capacity relative to the winter oilseed rape average. Among the three highest yielding ideotype plants, the number of secondary inflorescences is between 10 and 12, (compared to an average of 8 among winter oilseed rape), and more than 850 pods on secondary branches. This overall strategy is qualitatively similar to that of the single best performing observed plant.

### Trait-trait relationships exaggerate trait heritability estimates

When choosing traits for genetic selection, it is important to ascertain that genetically controlled variation in the trait exists within the available breeding material. However, we find that many of the measured traits can be well predicted from observations of their parent traits (S5 Fig). This implies that a large part of the observed variation in these traits may be caused indirectly, by physiological links to variation in their parent traits. As shown in S3 Table, the majority of measured traits exhibit evidence for substantial “total” broad sense heritability. However, when variation caused by parent traits is controlled for by using model residuals, estimates of “direct” heritability (in which genetic factors act directly on the trait, and not via its parent traits) can substantially decrease. For example, S3 Table shows that heritability for pod abortion on the main branch (%abortion M) drops by a factor of 3x when variation in its parent traits (number of flowers and number of secondary branches) are controlled for. This indicates that when attempting to produce a plant defined by multiple traits, care must be taken in trait selection, as there is less potential for their independent trait modification than might be indicated by “total” broad sense heritability.

### Trait-trait relationships can mask genetic associations

**Fig 7:**
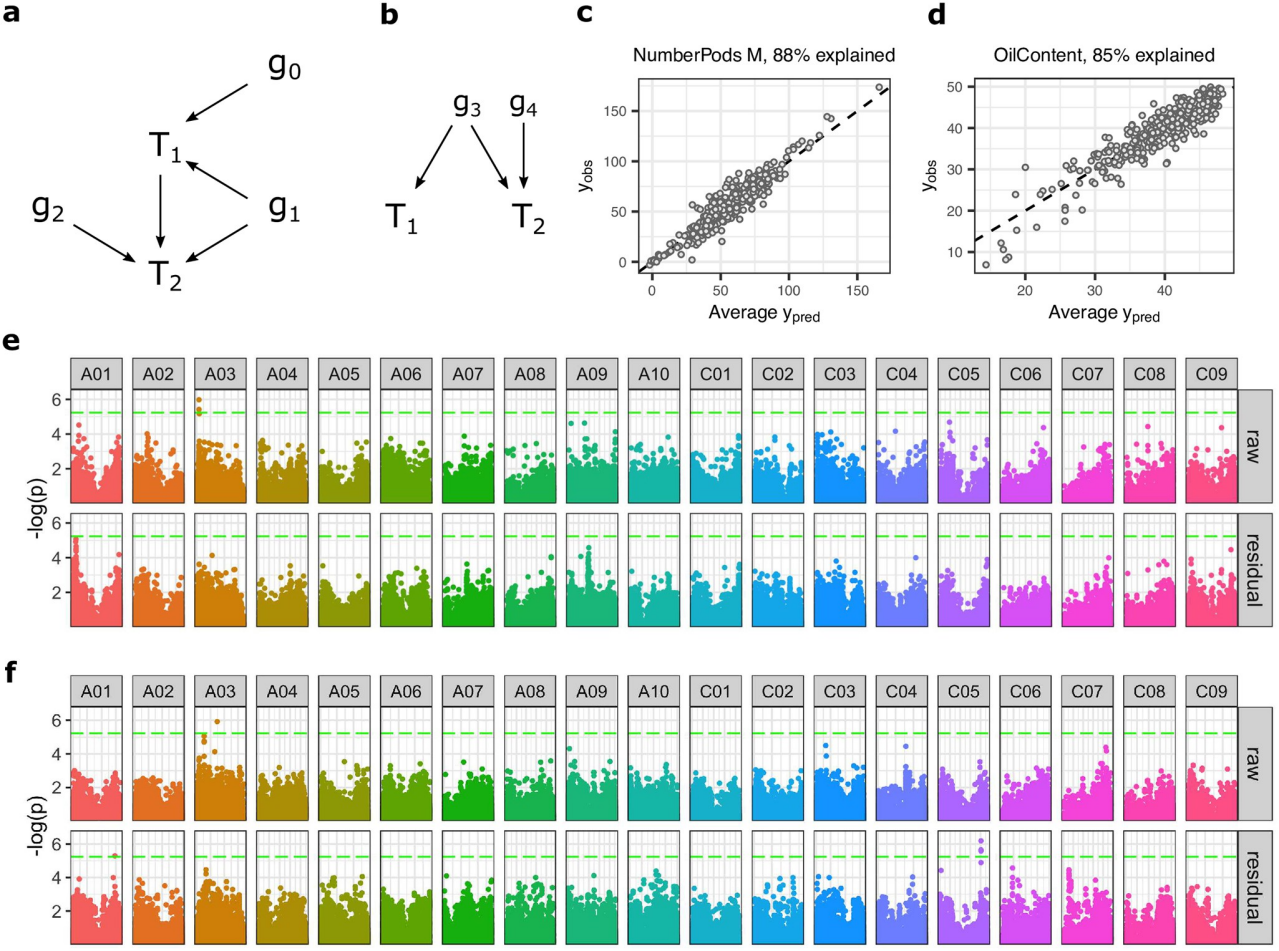
Accounting for trait-trait relationships in real data, can identify SNPs which likely act indirectly, and identify SNP associates which are missed when no correction for is applied. **a)** Causal diagram showing relationships between traits (T) and genes (g). If a direct causal relationship exists between traits, genes may affect a trait indirectly via another trait (g_0_, mediated pleiotropy), directly (g_2_), or via both of these mechanisms (g_1_). **b)** A directly causal trait-trait relationships may be erroneously inferred due to a confounding latent variable, for example a pleiotropic gene which causes both traits to be correlated (g_3_). **c)** Variation in the number of seed pods on the main inflorescence (NumberPods M), and **d)** seed oil content (OilContent) can be well explained by variation in their parent traits. Manhatten plots for SNPs associated to **e)** number of pods on the main inflorescence (NumberPods M), and **f)** seed oil content (OilContent). Association is tested either the raw trait (raw), or after correction for parent trait values (residual). Statistical significance is shown with Bonferroni corrected FWER = 0.1 (see Methods). For NumberPods M, a significant peak on chromosome A03 in the raw plots disappears in the residual plots. Assuming a causal relationship between the parent traits and NumberPods M, then this peak is likely caused by mediated pleiotropy. For seed oil content, a statistically significant peak appears on C05 in the residual plot. This is because accounting for variance caused by other factors increases the power to detect the genetic variant underlying the C05 peak. The factors accounted for may be the genes underlying the parent traits, (if there is a direct physiological link between the parent trait and oil content), in which case the C05 variant is similar to g_2_ in **a**. If instead, oil content and its parent traits have a common genetic cause then this can still result in increased power to detect genes affecting oil content only, by correcting for the variance introduced by these directly pleiotropic genes (by removing the effect of g_3_, g_4_ is more easily detected in **b**.

When traits can influence each other through some physiological link, a gene may have an indirect causal link to the (child) trait of interest, mediated by its direct effect on intermediate (parent) traits (Fig 7a). This is called mediated pleiotropy, and is distinct from biological pleiotropy in which a gene directly influences multiple traits (Fig 7b, Solovieff et al., 2013). One method to distinguish genes acting directly on a trait from genes acting through mediated pleiotropy is to correct for the association between causally linked traits using the residuals of modelled trait-trait relationships [34]. This eliminates association between the trait of interest and the parent trait(s), and therefore breaks the causal path between trait of interest, and any SNPs which affect it through the parent trait(s).

Under simulation, we find that when variation in the child trait is predominantly caused by variation in the parent trait (as appears to be the case for many of the experimentally observed traits, S5 Fig), then unless the correction is applied, SNPs which act on the parent are preferentially identified over SNPs which act directly on the child trait S6 Fig). Simulation also shows that correction for trait-trait relationships increases the power to detect directly causal SNPs (S6 Fig), as without correction, the child trait is effectively more genetically complex, being caused not only by the SNPs which affect it directly, but also by all the SNPs which effect the parent trait(s).

We also see evidence that correcting for trait-trait relationships by model residuals can identify mediated pleiotropy effects and increase the power to detect trait associated SNPs in our experimental data. We conducted GWAS analysis on traits measured in our oilseed rape panel, with and without correction for the effects of trait-trait relationships. Fig 7 shows GWAS results for number of pods on the main inflorescence, and seed oil content, both of which are well explained by their parent traits (Fig 7c and 7d). Fig 7e shows a statistically significant peak on chromosome A03 associated with the observed number of pods on the main inflorescence. However, this peak (which is visible when using the “raw” data) disappears when using the corrected “residual” data. Assuming a true underlying physiological trait-trait relationship exists between the number of pods on the main inflorescence and its parent traits (number of secondary inflorescences, height, number of flowers, and % pod abortion), then the disappearance of a peak in the raw observation data indicates that the causal mutation under the peak on A03 pod number indirectly via mediated pleiotropy, through its effect on a parent trait (as g_0_ in Fig 7a).

Fig 7f shows SNP association with seed oil content. A statistically significant peak on chromosome C05 appears in the residuals Manhattan plot, after the trait relationship correction is applied. This corresponds to a QTL region previously described as affecting oil content in *Brassica napus* [59, 60]. The detection of this peak using residual correction is due to the increased power to detect the underlying SNP’s association to oil content after other factors associated with oil content are accounted for. The other factors may be a directly causal physiological interaction between oil content and its parent traits (in which case the gene under the C05 peak is analogous to g_2_ in Fig 7a), or underlying genetic factors which directly affect both oil content and its parent traits (Fig 7b). The genetic factors affecting both traits may either be true biologically pleiotropic genes, or alleles in different genes which are correlated in the population (either through linkage disequilibria, or through joint selection). In this example, it is likely that both mechanisms are relevant: as well as a plausible causal link between the traits themselves, seed yield and oil content are under joint selection (both are positive traits for oilseed rape breeding programmes), and so alleles affecting these traits are likely to be correlated in the population.

Using models of trait-trait relationships, we gained statistical power, allowing detection of interesting SNP associations in previously reported QTL regions. We were also able to differentiate between direct SNP associations with the trait of interest, and mediated pleiotropy, avoiding the potentially misleading identification of genes which act indirectly through parent traits.

## Discussion

Here, we have analysed the underexplored consequences of trait-trait relationships, finding that they influence key yield traits and ideotypes, heritability estimates, and gene-trait association studies. The individual approaches have been described previously but the combination presented here provides a novel, general methodological framework for the study of crop breeding as an optimisation problem, accounting for causal trait-trait relationships. With phenomics platforms increasingly enabling the observation of large numbers of potentially related traits [61], we consider a computational framework for handling such datasets to be a timely development.

We inferred the trait network structures that are most parsimoniously able to explain the empirically observed trait-trait relationships, consistent with prior beliefs about trait relationships. Sequential path analysis may be viewed as an intermediate between statistical yield modelling (in which a trait’s relationship with yield is inferred from the data only) and mechanistic crop models (in which a biological understanding of the system is used to define the model structure and trait-trait relationship functions *a priori*). Highly connected networks were inferred for both spring and winter oilseed rape, supporting the view many yield traits affect each other.

The inferred trait models were used to predict the impact of changing each trait on seed yield in spring and winter oilseed rape, accounting for compensatory or exacerbating changes in other traits. Similar results were found for spring and winter oilseed rape. Interestingly we find that many previously identified yield traits, including traits which are correlated with yield in our dataset, are not predicted to affect seed yield if changed. This is perhaps surprising but reflects the many compensatory and buffering relationships between traits in the developmentally plastic oilseed rape plant. By observing multiple traits simultaneously, we were able to identify trait-trait relationships which result in misleading correlations or lack of correlation between individual traits and yield. The only traits whose modification was confidently predicted to lead to variation in seed yield were the number of secondary inflorescences (NumberSecondInfl), pod length (PodLength), total seed number (SeedNumber), seeds per pod (SNPP), seed area (SeedArea) and thousand grain weight (TGW).

Identification of the individual traits whose modification is expected to alter seed yield ignores potential interactions between traits in the function mapping traits to yield. We therefore identified the points in the multidimensional trait space which maximise the expected improvement in seed yield over the best observed yield in each of the spring and winter oilseed rape panels. The identified points define “ideotypes”—hypothetical plants which are expected to produce high seed yield based on a model of the way that yield traits interact to produce seed yield.

In spring oilseed rape, we found that when observed trait trade-offs were imposed as constraints, many of the observed plants were higher yielding than the best ideotypes identified (which are required to be unlike observed plants). This implies that the high yielding regions of trait space are already well explored by the existing varieties, and it is not expected that other plants can be defined in terms of these experimentally observed traits, which are likely to yield better under these growing conditions. Therefore, our modelling suggests that under these conditions, spring oilseed rape plants should ideally produce 35 to 60 pods on the main branch, approximately 150 to 250 pods should be produced on secondary branches, seed number should be > 4000, seed weight per 10 pods on the main branch should be > 0.6g, and TGW should be approximately 3g to 5g. In winter oilseed rape, ideotypes were identified which are expected on average to perform better than all but the single best observed plant. This suggests that regions of the trait space exist, which are likely to yield better than the existing varieties when grown under these conditions. This difference between spring and winter oilseed rape may reflect greenhouse conditions being more like the conditions that spring oilseed rape has been selected for. The best identified winter oilseed rape ideotype has 90 pods on the main branch, and approximately 900 pods across 11 secondary branches. It produces just under 6000 seeds in total, with a relatively small TGW of 4g. ovule area, and seed weight per 10 pods on the main branch is approximately average for winter varieties. Interestingly, many optimal trait values are intermediate rather than extremely high or low. This is consistent with the largely non-linear, saturation type relationships individual traits exhibit with yield. These results suggest that the linear models widely used in yield-trait association modelling may be misleading in recommending selection either for or against a particular trait to improve seed yield. Instead, for the majority of traits an optimal region exists, with suboptimal regions both above and below it.

In identifying ideotypes through optimisation, we have constrained correlation relationships among yield traits to be the same as those observed in the experimental plants. This assumes that the observed relationships have unavoidable, physiological causes (such as competition between traits for seed filling capacity) rather than correlated underlying genetic causes which could potentially be broken through selection. We have shown that if the observed correlations between traits can be broken, then large increases in potential seed yield may be possible. However, it is doubtful that the key relationships can be relaxed, as it is generally considered that in oilseed rape seed number and seed size directly compete for seed filling resources [1].

The importance of different traits in determining yield varies with environment (S7 Fig). Here, the calculated ideotypes were generated from data collected under greenhouse conditions, and the ideotype trait values obtained are expected to be specific to these conditions. Trait-trait models should be developed under the environmental conditions of interest, for the suggested EI optimisation to provide the best recommendations for those conditions. The identified ideotypes are defined in terms of multiple desired trait values, however this does not address whether these ideotypes can be produced through selective breeding. High heritability is required for genetic selection, and as shown here, causal trait-trait relationships mean that often a large proportion of a trait’s heritability is not due to genetic variation in the trait itself, but due to genetic variation in its ancestor traits within the trait-trait relationship DAG. Consequently, each trait’s heritability independent of the other traits is lower than would be calculated for each individually. Therefore, it is recommended that given a list of multiple candidate traits for selection within a particular programme, residual heritability for each trait (conditioned upon the others) be calculated to estimate the extent to which the desired trait values can be mutually selected for.

Yield is a complex trait and its decomposition into contributing yield traits leads to non-linear relationships between these yield traits. Ideally, we would be able to infer associations between genetic variants and seed yield directly from available data. However, we have shown that causal associations between traits mean that genes which affect each trait directly also affect its descendent traits indirectly. This results in accumulating genetic complexity as the trait hierarchy is descended, and consequently relatively large sample sizes are required to provide the statistical power to identify genetic variants which affect yield as a relatively large numbers of genes with relatively small effects can be expected compared to upstream traits. By factorising the effect of the genome on yield into its effects on each upstream yield trait, the problem is simplified, and association can be detected through less statistically powerful experiments assuming known trait-trait relationships. As we have seen, many other traits in oilseed rape besides yield are also “complex” (in that multiple ancestor traits contribute to their value) and can also benefit from a decomposition approach. By testing for association to the residuals of a trait-trait model, rather than to observed traits directly, the effects of trait-trait relationships are controlled for, and the undesirable accumulation of genetic complexity can be avoided. Additionally, by using the modelled trait-trait relationships to identify mediated pleiotropy, genes directly regulating the trait of interest can be identified, rather than its parent traits.

The potential benefit of correcting for causal trait-trait relationships depends on the relative contribution of direct, physiologically caused trait relationships over classical pleiotropic gene action, in which a gene independently influences two traits. Whether a link is directly causal (rather than due to a confounding latent variable) cannot be distinguished without either perturbation experiments or making the uncounfoundedness assumption—that all relevant variables are observed and included in the model. The requisite perturbation experiments generally have not been performed within the context of yield trait association studies [9, 12, 22, 24, 36, 37, 62–70]. It is therefore not shown that all the links inferred in the model are directly causal. Linked traits may instead have a confounding common cause. Either an unobserved third trait, a shared pleiotropic gene, or a set of genes which act on the traits independently, but which are correlated in the population. In this study, we have largely assumed that the trait-trait relationships inferred are direct, causal relationships, caused by physiological links, in order to allow a thought experiment into the consequences of these relationships, and highlight the importance of their consideration. This is reasonable in many cases, either due to known developmental associations, physiological feedback regulating traits, or competition for resources between observed traits. The identified links therefore provide a set of hypothetical relationships consistent with the observed data and prior information, and which should be experimentally verified in an iterative process of model improvement.

Overall, we have shown the importance of accounting for trait-trait relationships in many facets of applied plant science, from identifying crop ideotypes, to estimating which traits can be selected for, to identifying gene-trait association. Taking these relationships into account is becoming increasingly tractable due to the introduction of phenomics platforms, allowing the simultaneous measurement of numerous traits. The analysis presented here exemplifies a novel methodological framework to make best use of this emerging experimental design.

## Supporting information

S1 Fig Oilseed rape varieties group by type in PCA of varieties used for GWAS. Principal component analysis was carried out using the 108,653 SNPs detected by Havlickova et al., 2017, to check that the identified SNPs reflect the expected population structure.
(TIF)

S2 Fig QQ-plots for models to associate SNP data with the number of pods on the main inflorescence (NumberPods M), and seed oil content (OilContent). QQ plots are shown for models associating SNPs to the trait directly (raw), and to the residuals of models in which the trait is predicted from its parent traits.GLM-Q, GLM-PCA5d, GLM-PCA10d are general linear models (GLM), which use the SNP data, as well as either the population structure matrix (Q), first five (PCA5d), or first ten principle components (PCA10d) of the SNP matrix. MLM-K, MLM-Q+K, MLM-PCA5d+K are mixed linear models (MLM) which use the SNP data, as well as the Q-matrix, PCA components, or kinship matrix (K). A Bayesian-information and Linkage-disequilibrium Iteratively Nested Keyway (BLINK) model was fit using the GAPIT package implementation. Based on these plots, GLM-PCA5d was used for NumberPods M, and GLM-PCA10d was used for OilContent.
(TIF)

S3 Fig Traits may be correlated to seed yield without a causal relationship between them. Predicted trait values (y-axis) as traits a singly varied (x-axis). Median prediction shown with a heavy line, confidence quantiles are shown with 10% intervals. The predicted effect of modifying **a)** silique beak length, **b)** pod length on other yield traits and seed yield in Winter oilseed rape. Changing beak length is not expected to much affect yield. Modifying pod length is expected to strongly affect both beak length and seed yield. Consequently beak length and seed yield are correlated. **c)** a trade-off exists between seed number and thousand grain weight (TGW) in both Spring and Winter oilseed rape.
(TIF)

S4 Fig Oilseed rape ideotypes identified ignoring empirically observed relationships between traits. Facets show traits which affect seed yield directly in the **a)** Spring and **b)** Winter oilseed rape panels. Coloured points show mean predicted seed yield for identified hypothetical ideotype plants with the indicated yield trait values. Their order reflects their probability of having greater seed yield than the best experimentally observed plant, with 1 having the best chance, and 10 the worst of the calculated ideotypes. These points are identified through Bayesian optimisation as the unobserved points with the best chance of exceeding the maximum observed seed yield in each panel. Thin black line shows the maximum observed seed yield produced by any of the plants in the experimental panels, thick black line shows mean observed seed yield. Black dashes show yield trait values for experimentally observed plants. If expected improvement in seed yield is maximised without the constraint of respecting observed correlations between yield traits, then more optimal regions of trait space exist than the regions occupied by observed plants for both spring and winter oilseed rape. As might be expected, this is largely via breaking the negative trade-off between seed size, and seed number in both Spring and Winter oilseed rape.
(TIF)

S5 Fig Many yield traits can be well predicted from their parent traits. Plots show the mean predicted trait values vs observed trait values for each plant in **a)** Spring oilseed rape, **b)** Winter oilseed rape. Variance in the trait explained by the parents is given above each plot. It can be seen that variation in many traits can be well explained by their parent traits, indicating that this variation may not be due to direct genetic or stochastic variation in the trait itself, but instead due to variation in its parent traits.
(TIF)

S6 Fig Simulation shows that unless accounted for, trait-trait relationships reduce power to detect gene associations as well as the misleading identification of indirectly associated genes. **a)** Model from which simulated data were generated for each plant independently (j). Parent-trait (p) is the weighted sum of “parent SNPs” (s_1_, … s_n_) which affect it directly. Child-trait (c) is the weighted sum of “child SNPs” (r_1_, …, r_m_), as well as the parent trait. So, it is affected directly by the child SNPs, and indirectly by the parent SNPs. Parent SNPs will therefore exhibit mediated pleiotropy. Noise (*ϵ*^*p*^, *ϵ*^*c*^) was added to both traits. “Non-causal SNPs” (q_1_, …, q_k_) do not affect either trait. All SNPs were independently sampled from a Bernoulli(0.5) distribution. **b)** The number of “parent SNPs”, “child SNPs” and “non-causal SNPs” statistically associated with variation in the child-trait, using either observations of the child trait directly (red), or correcting for trait-trait relationships, by using the residuals of a model in which child-trait was predicted by parent-trait (blue). (See methods section for details of associated SNP inference). In all tested cases, (except when c is independent of p, *γ* = 0), the power to detect child SNPs is greater when the effect of p on c is controlled for. The greater the value of *γ*, the bigger the difference. When *γ* is large relative to *σ*, parent SNPs are identified rather than child SNPs due their indirect effect (for example when *γ* = 3, *σ* = 0.1). When *γ* is similar to *σ*, association using the direct observations of c was less able to detect any directly or indirectly causal SNPs (for example when *γ* = 1, *σ* = 0.5). Neither method was more associated with spurious identification of non-causal SNPs.
(TIF)

S7 Fig Correlations between traits and yield vary based on environment, and varieties considered. The measures of yield shown are “SeedYield” (weight of seed produced per plant), or “SeedYield (kg / hectare)”. Lu et al, Jeromela et al, Aytac & Kinaci 2003 & 2004 use Winter oilseed rape panels. Chen et al use Spring and Winter oilseed rape, the remainder use Spring oilseed rape panels. Years indicate repeated trials in the same study, with the exception of Khan 2000, Khan 2006 which are separate studies. Sabaghnia et al., and Ivanovska et al., alter environmental conditions within a study, either experimentally or through trial location. Referenced studies are [12, 22, 24, 36, 37, 62–68, 70].
(TIF)

S1 Table Macro- and microtraits. List of macrotrait (n = 5) and microtrait (n = 3) names and abbreviations measured in the diversity set population.
(DOCX)

S2 Table Normalising transformations. List of transformations applied to normalise trait distributions. Id = identical (no transformation applied).
(DOCX)

S3 TableEstimated genetic control of phenotypic traits.Statistical significance of genotype effect on measured traits estimated by one way ANOVA. Reported p-values were adjusted for multiple hypothesis testing by Benjamini-Hochberg method. Broad sense heritability ($H^{2}=\sigma_{g}^{2}/\sigma_{p}^{2}$) was estimated from the mean squares components of ANOVA, following [28], using either normalised observed trait values, or model residuals (see methods). Dashes indicate that the trait is not modelled as having any parent traits, and so residual values are the same as for raw values.(DOCX)

S1 File List of 94 varieties included in the oilseed rape diversity set population. The ASSYST code, genotype names, crop type description and the 4 oilseed rape groups are presented.
(XLSX)

S2 File Trait-trait relationship constraints for sequential path analysis. (XLSX)

S3 File Supplemental methods. Heritability estimation and causal SNP identification.
(DOCX)
