# Supplementary figures and images for: A causal inference and Bayesian optimisation framework for modelling multi-trait relationships—Proof-of-concept using Brassica napus seed yield under controlled conditions
Source: PLoS One. 2023 Sep 1;18(9):e0290429. doi: 10.1371/journal.pone.0290429 (PMC10473526; doi:10.1371/journal.pone.0290429)

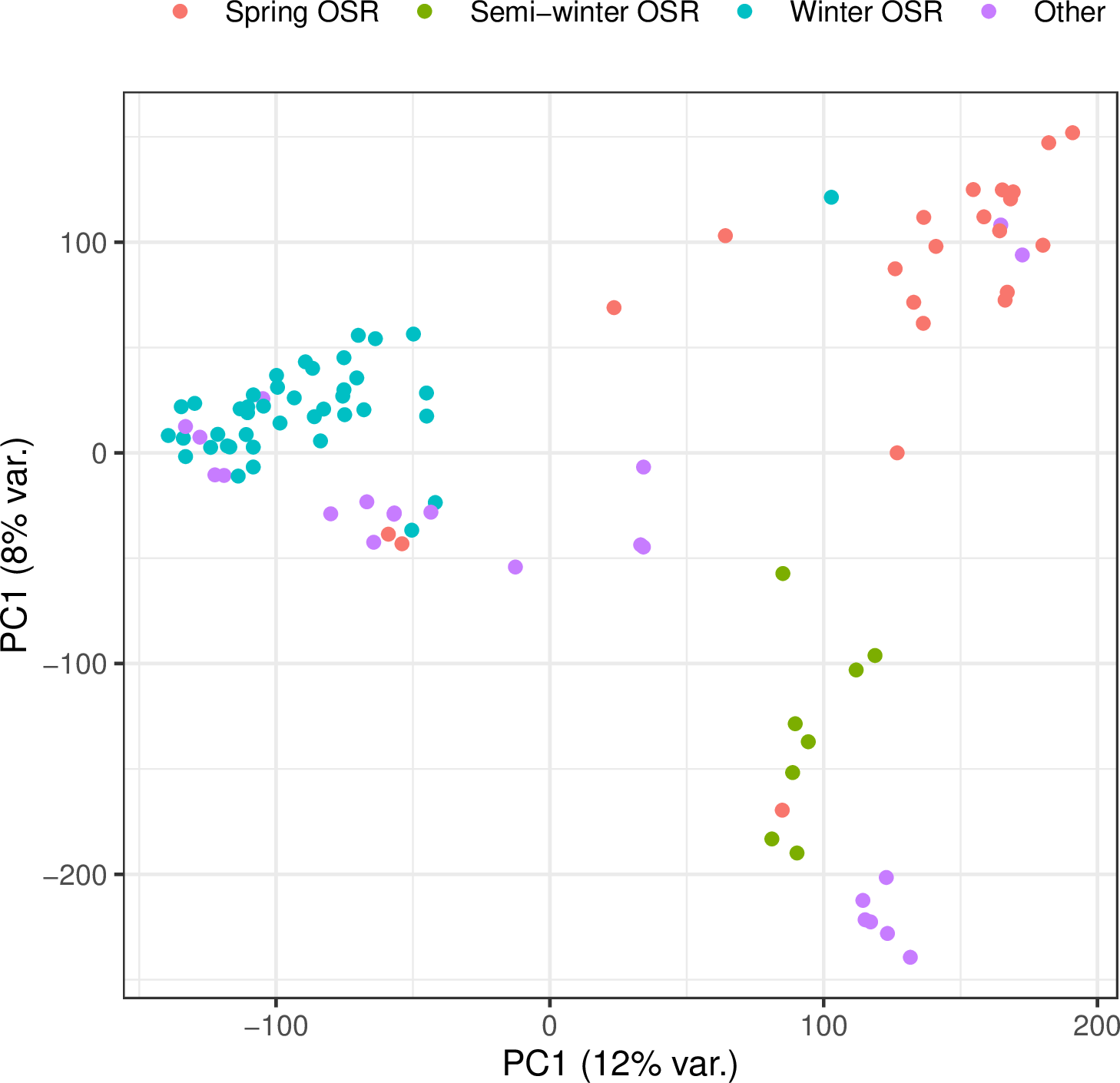

Supplement: S1 Fig — Principal component analysis was carried out using the 108,653 SNPs detected by Havlickova et al., 2017, to check that the identified SNPs reflect the expected population structure. (TIF) [file pone.0290429.s001.tif]

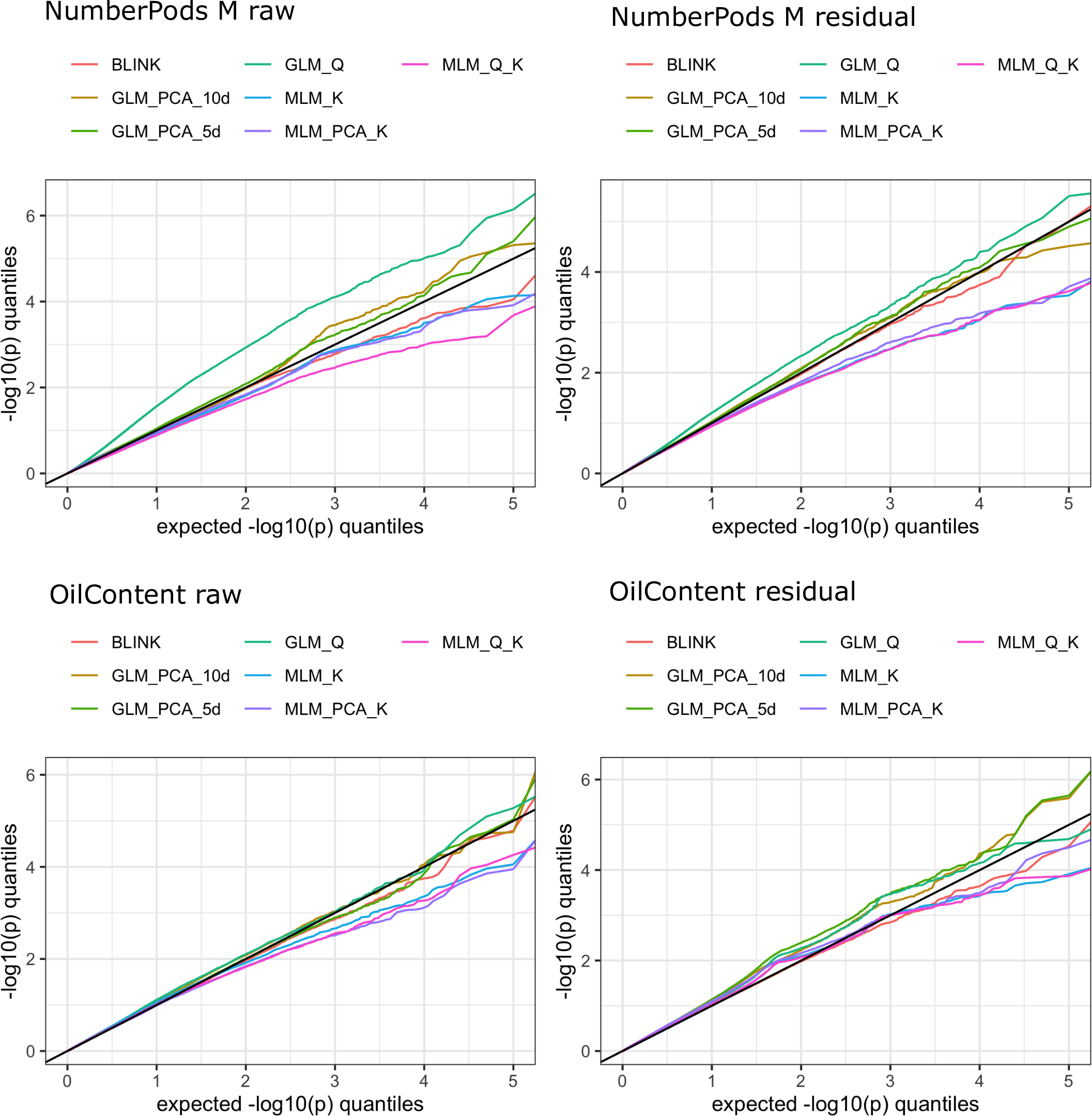

Supplement: S2 Fig — QQ plots are shown for models associating SNPs to the trait directly (raw), and to the residuals of models in which the trait is predicted from its parent traits.GLM-Q, GLM-PCA5d, GLM-PCA10d are general linear models (GLM), which use the SNP data, as well as either the population structure matrix (Q), first five (PCA5d), or first ten principle components (PCA10d) of the SNP matrix. MLM-K, MLM-Q+K, MLM-PCA5d+K are mixed linear models (MLM) which use the SNP data, as well as the Q-matrix, PCA components, or kinship matrix (K). A Bayesian-information and Linkage-disequilibrium Iteratively Nested Keyway (BLINK) model was fit using the GAPIT package implementation. Based on these plots, GLM-PCA5d was used for NumberPods M, and GLM-PCA10d was used for OilContent. (TIF) [file pone.0290429.s002.tif]

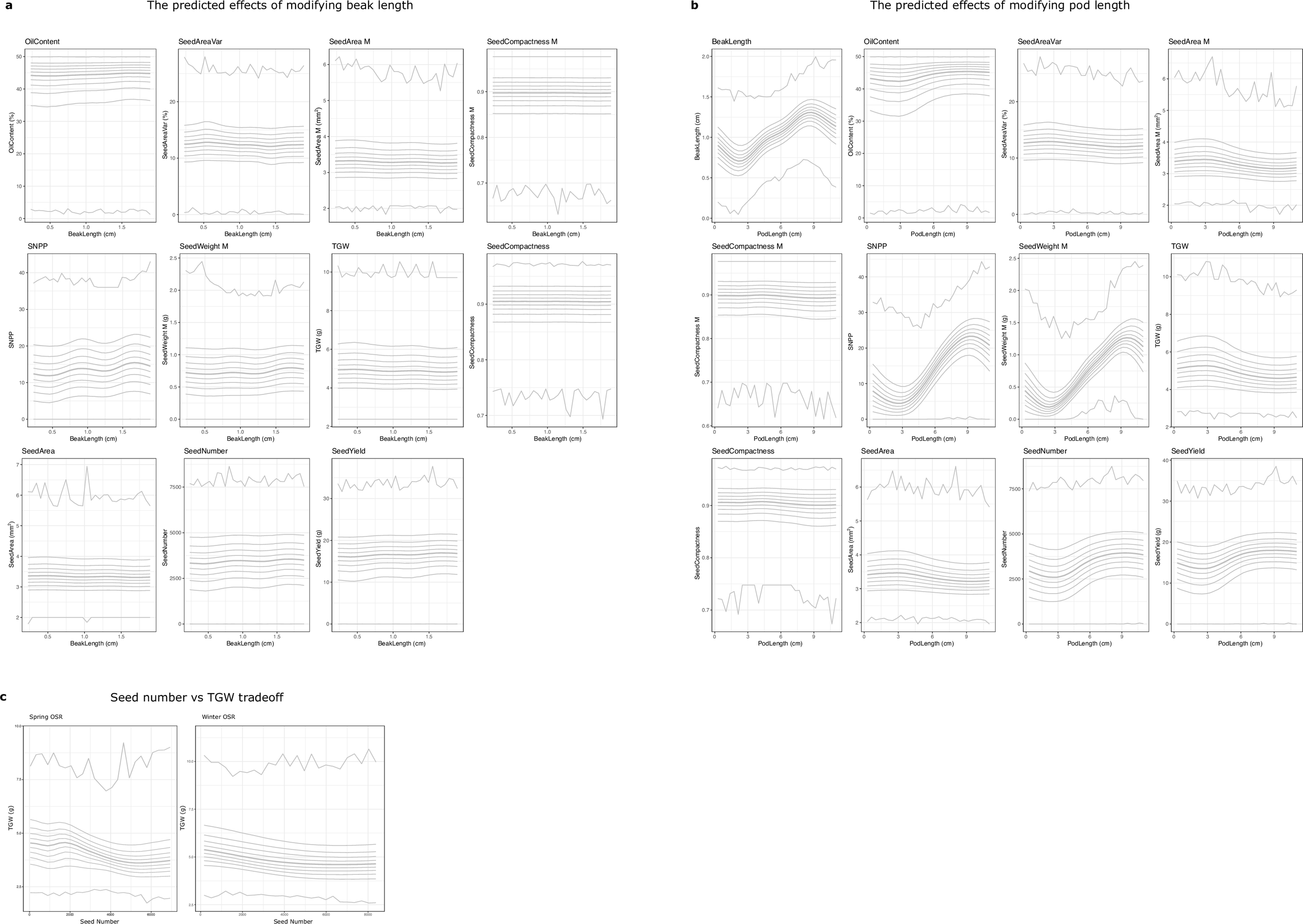

Supplement: S3 Fig — Predicted trait values (y-axis) as traits a singly varied (x-axis). Median prediction shown with a heavy line, confidence quantiles are shown with 10% intervals. The predicted effect of modifying a) silique beak length, b) pod length on other yield traits and seed yield in Winter oilseed rape. Changing beak length is not expected to much affect yield. Modifying pod length is expected to strongly affect both beak length and seed yield. Consequently beak length and seed yield are correlated. c) a trade-off exists between seed number and thousand grain weight (TGW) in both Spring and Winter oilseed rape. (TIF) [file pone.0290429.s003.tif]

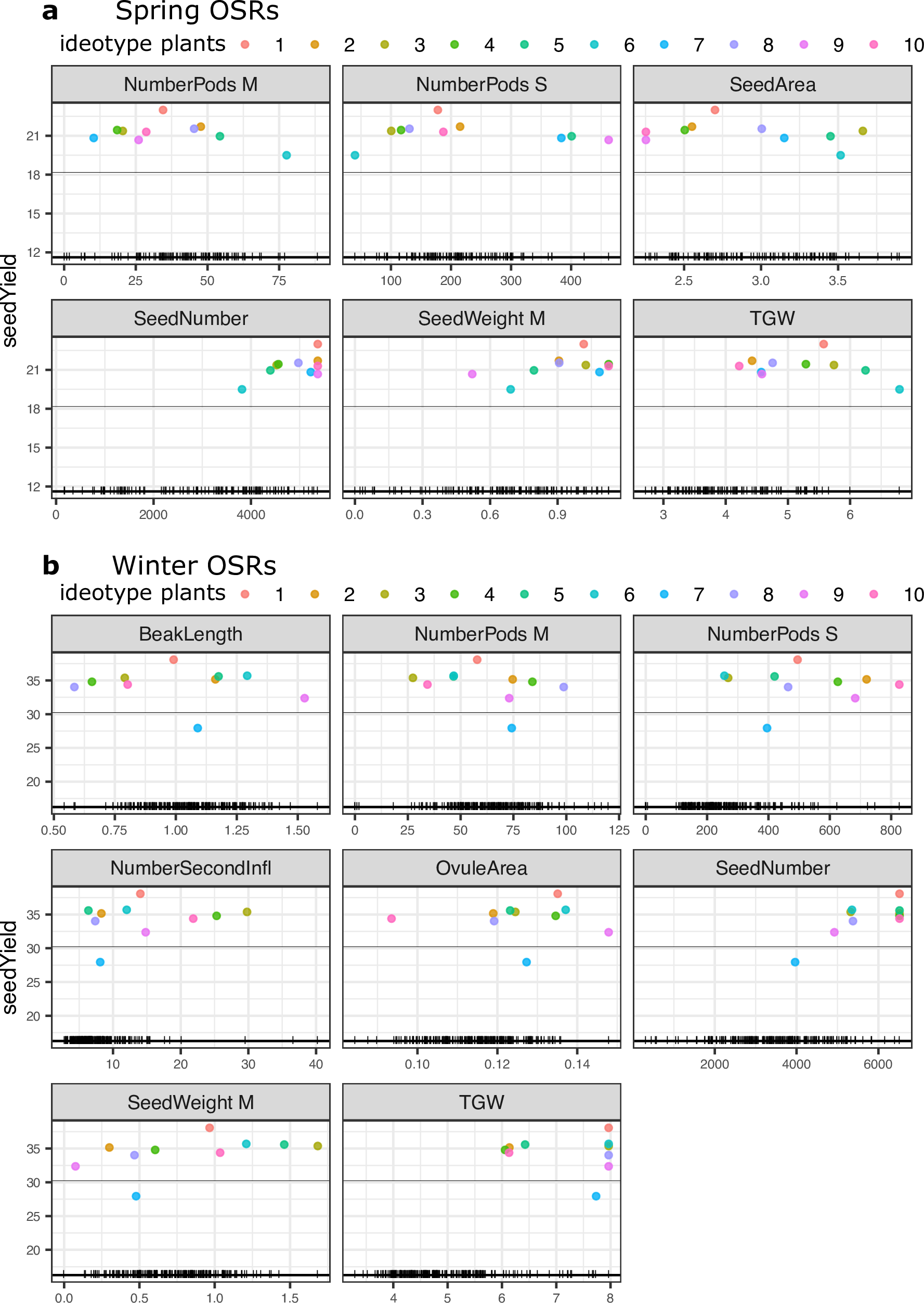

Supplement: S4 Fig — Facets show traits which affect seed yield directly in the a) Spring and b) Winter oilseed rape panels. Coloured points show mean predicted seed yield for identified hypothetical ideotype plants with the indicated yield trait values. Their order reflects their probability of having greater seed yield than the best experimentally observed plant, with 1 having the best chance, and 10 the worst of the calculated ideotypes. These points are identified through Bayesian optimisation as the unobserved points with the best chance of exceeding the maximum observed seed yield in each panel. Thin black line shows the maximum observed seed yield produced by any of the plants in the experimental panels, thick black line shows mean observed seed yield. Black dashes show yield trait values for experimentally observed plants. If expected improvement in seed yield is maximised without the constraint of respecting observed correlations between yield traits, then more optimal regions of trait space exist than the regions occupied by observed plants for both spring and winter oilseed rape. As might be expected, this is largely via breaking the negative trade-off between seed size, and seed number in both Spring and Winter oilseed rape. (TIF) [file pone.0290429.s004.tif]

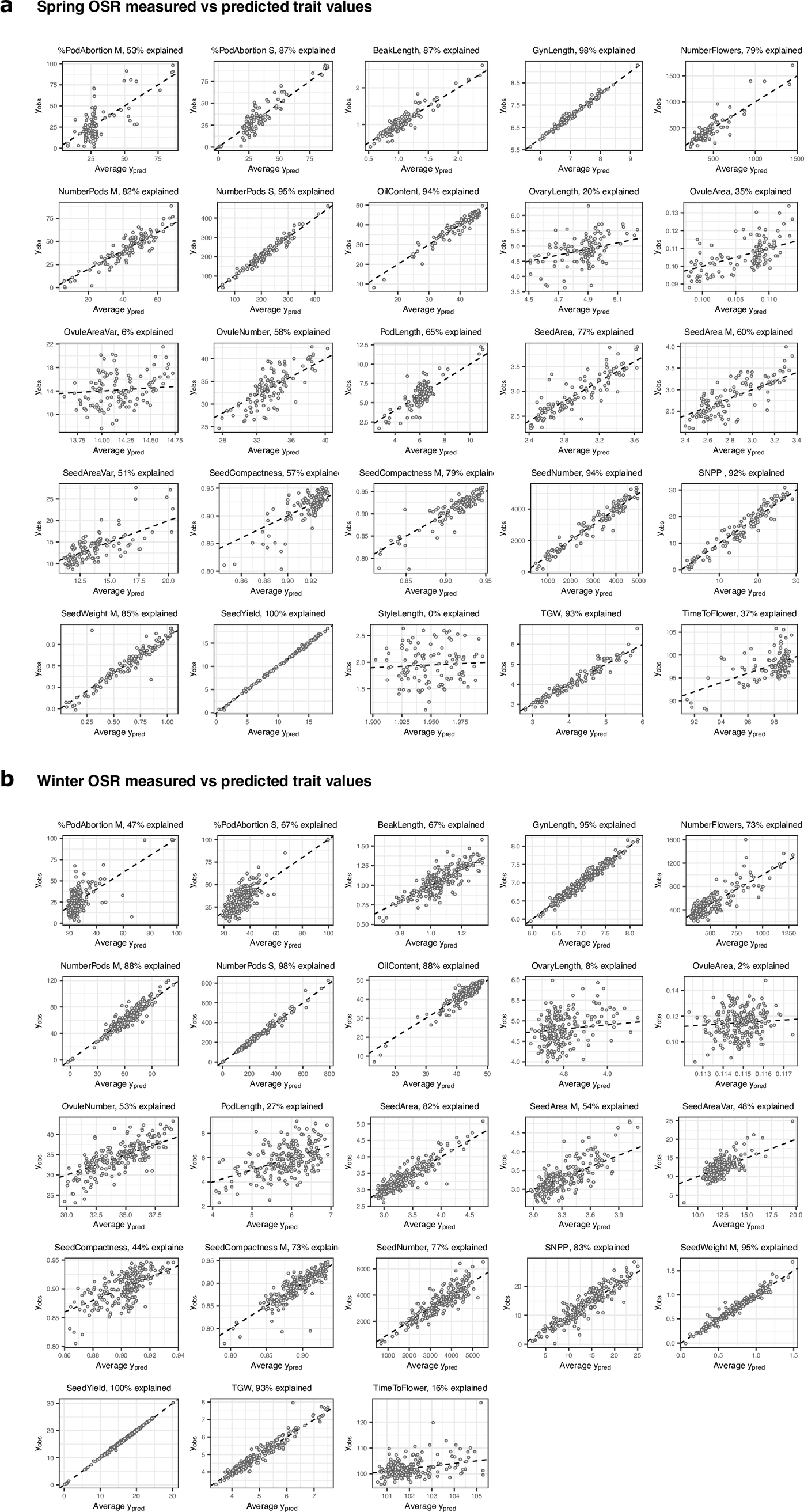

Supplement: S5 Fig — Plots show the mean predicted trait values vs observed trait values for each plant in a) Spring oilseed rape, b) Winter oilseed rape. Variance in the trait explained by the parents is given above each plot. It can be seen that variation in many traits can be well explained by their parent traits, indicating that this variation may not be due to direct genetic or stochastic variation in the trait itself, but instead due to variation in its parent traits. (TIF) [file pone.0290429.s005.tif]

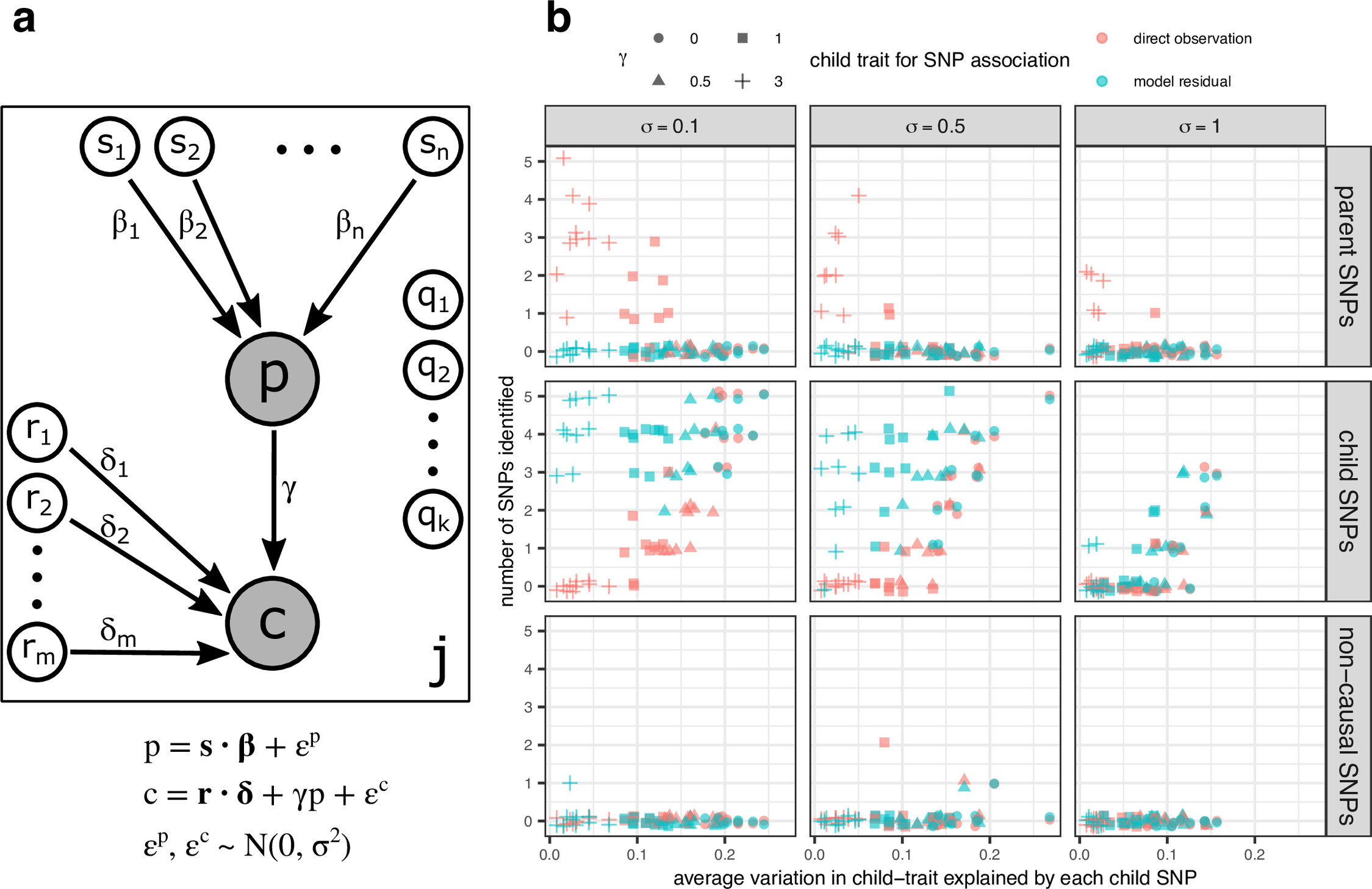

Supplement: S6 Fig — a) Model from which simulated data were generated for each plant independently (j). Parent-trait (p) is the weighted sum of “parent SNPs” (s1, … sn) which affect it directly. Child-trait (c) is the weighted sum of “child SNPs” (r1, …, rm), as well as the parent trait. So, it is affected directly by the child SNPs, and indirectly by the parent SNPs. Parent SNPs will therefore exhibit mediated pleiotropy. Noise (ϵp, ϵc) was added to both traits. “Non-causal SNPs” (q1, …, qk) do not affect either trait. All SNPs were independently sampled from a Bernoulli(0.5) distribution. b) The number of “parent SNPs”, “child SNPs” and “non-causal SNPs” statistically associated with variation in the child-trait, using either observations of the child trait directly (red), or correcting for trait-trait relationships, by using the residuals of a model in which child-trait was predicted by parent-trait (blue). (See methods section for details of associated SNP inference). In all tested cases, (except when c is independent of p, γ = 0), the power to detect child SNPs is greater when the effect of p on c is controlled for. The greater the value of γ, the bigger the difference. When γ is large relative to σ, parent SNPs are identified rather than child SNPs due their indirect effect (for example when γ = 3, σ = 0.1). When γ is similar to σ, association using the direct observations of c was less able to detect any directly or indirectly causal SNPs (for example when γ = 1, σ = 0.5). Neither method was more associated with spurious identification of non-causal SNPs. (TIF) [file pone.0290429.s006.tif]

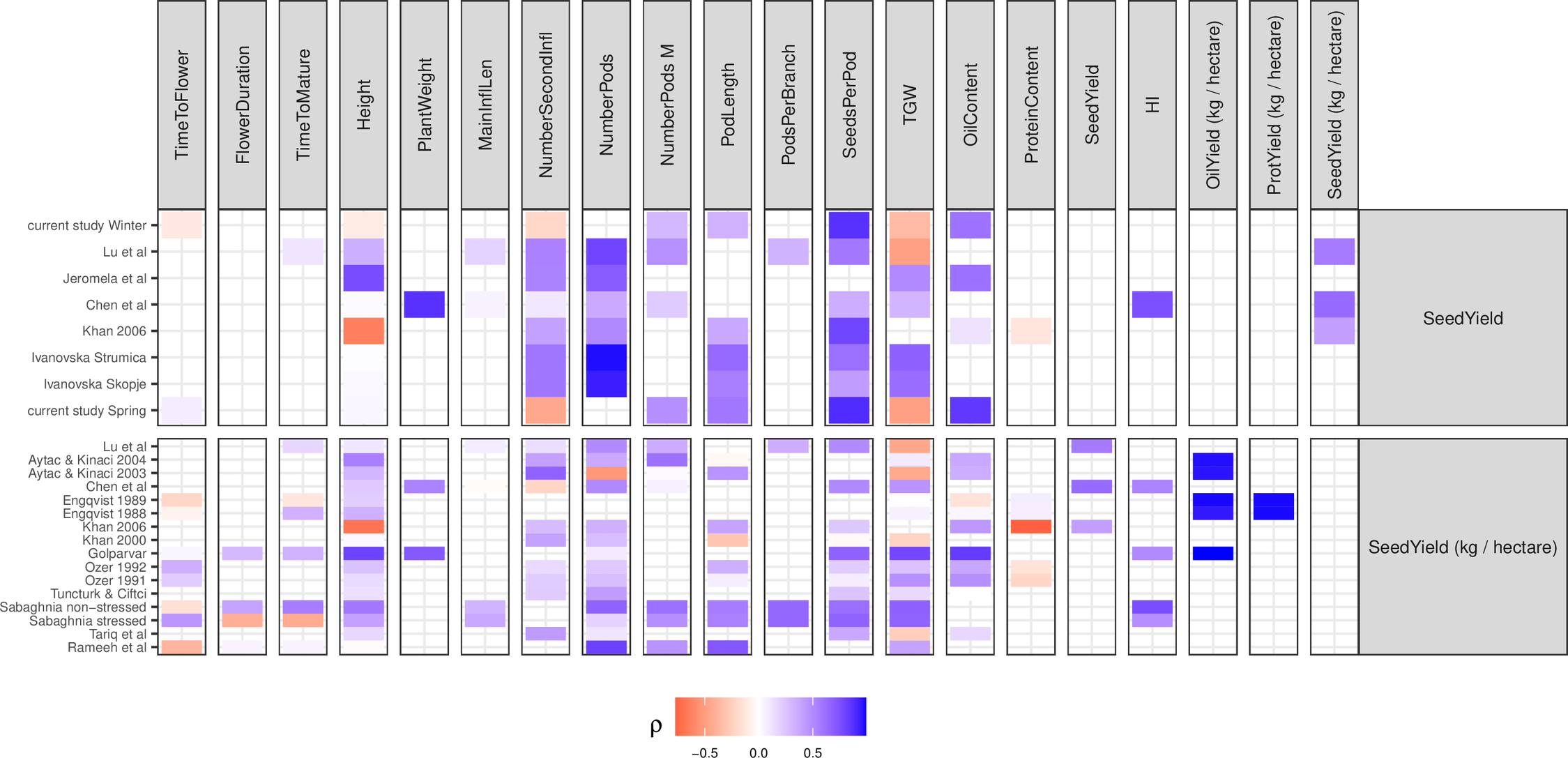

Supplement: S7 Fig — The measures of yield shown are “SeedYield” (weight of seed produced per plant), or “SeedYield (kg / hectare)”. Lu et al, Jeromela et al, Aytac & Kinaci 2003 & 2004 use Winter oilseed rape panels. Chen et al use Spring and Winter oilseed rape, the remainder use Spring oilseed rape panels. Years indicate repeated trials in the same study, with the exception of Khan 2000, Khan 2006 which are separate studies. Sabaghnia et al., and Ivanovska et al., alter environmental conditions within a study, either experimentally or through trial location. Referenced studies are [12, 22, 24, 36, 37, 62–68, 70]. (TIF) [file pone.0290429.s007.tif]
