# Supplementary material for: A causal inference and Bayesian optimisation framework for modelling multi-trait relationships—Proof-of-concept using Brassica napus seed yield under controlled conditions
Source: PLoS One. 2023 Sep 1;18(9):e0290429. doi: 10.1371/journal.pone.0290429 (PMC10473526; doi:10.1371/journal.pone.0290429)
Supplement: S1 Table — List of macrotrait (n = 5) and microtrait (n = 3) names and abbreviations measured in the diversity set population. (DOCX) [file pone.0290429.s008.docx]

| Macrotraits | | Microtraits | |
| --- | --- | --- | --- |
| Trait name | **Abbreviation** | **Trait name** | **Abbreviation** |
| Plant height (cm) | Height | Ovule number | OvuleNumber |
| Number of secondary inflorescences | NumberSecondInfl | Ovule area (mm^2^) | OvuleArea |
| Time to flowering (days) | TimeToFlower | Ovary length (mm) | OvaryLength |
| Number of flowers on the whole plant | NumberFlowers | Gynoecia length (mm) | GynLength |
| Number of pods on the main inflorescence | NumberPodsM | Style length (mm) | StyleLength |
| Number of pods on secondary inflorescences | NumberPodsS | Beak length (cm) | BeakLength |
| Pod abortion on the main inflorescence (%) | %PodAbortionM | Ovule area coefficient of variation (%) | OvuleAreaVar |
| Pod abortion in secondary inflorescences (%) | %PodAbortionS |  |  |
| Pod length from 10 pods from the main inflorescence (cm) | PodLength |  |  |
| Seed number/ pod from 10 pods from the main inflorescence | SeedNumberM |  |  |
| Seed area from 10 pods from the main inflorescence (mm^2^) | SeedAreaM |  |  |
| Seed compactness from 10 pods from the main inflorescence | SeedCompactnessM |  |  |
| Seed weight from 10 pods from the main inflorescence (g) | SeedWeightM |  |  |
| Seed area from the whole plant (mm^2^) | SeedArea |  |  |
| Seed compactness from the whole plant | SeedCompactness |  |  |
| Seed area coefficient of variation from whole plant (%) | SeedAreaVar |  |  |
| Thousand grain weight (g) | TGW |  |  |
| Estimated total seed number from the whole plant (by TGW) | SeedNumber |  |  |
| Seed oil content from the whole plant (%) | OilContent |  |  |
| Seed weight from the whole plant (seed yield, g) | SeedYield |  |  |

**Supplemental Table 1:** List of macrotrait (n=5) and microtrait (n=3) names and abbreviations measured in the diversity set population.
