# Supplementary material for: A causal inference and Bayesian optimisation framework for modelling multi-trait relationships—Proof-of-concept using Brassica napus seed yield under controlled conditions
Source: PLoS One. 2023 Sep 1;18(9):e0290429. doi: 10.1371/journal.pone.0290429 (PMC10473526; doi:10.1371/journal.pone.0290429)
Supplement: S2 Table — List of transformations applied to normalise trait distributions. Id = identical (no transformation applied). (DOCX) [file pone.0290429.s009.docx]

| **Trait** | **Transformation** | **Detail** |
| --- | --- | --- |
| Height | square root |  |
| NumberSecondInfl | natural log |  |
| NumberFlowers | natural log |  |
| TimeToFlower | id |  |
| OvaryLength | natural log |  |
| StyleLength | id |  |
| OvuleNumber | id |  |
| OvuleArea | id |  |
| OvuleAreaVar | id |  |
| GynLength | id |  |
| %PodAbortion M | logit with offset | p=(x+1) / (tot + 2) log(p/(1-p)) |
| NumberPods M | id |  |
| PodLength | id |  |
| BeakLength | id |  |
| NumberPods S | square root |  |
| %PodAbortion S | logit with offset | p=(x+1) / (tot + 2) log(p/(1-p)) |
| SeedsPerPod M | id |  |
| Seed Area M | natural log |  |
| SeedWeight M | id |  |
| SeedArea | natural log |  |
| SeedNumber | natural log |  |
| SeedAreaVar | id |  |
| SeedCompactness | logit | log(x/(1-x)) |
| SeedCompactness M | logit | log(x/(1-x)) |
| TGW | id |  |
| OilContent | logit with reference of 50 | log(x/(50-x)) |
| SeedYield | id |  |

**Supplemental Table 2:** List of transformations applied to normalise trait distributions. Id= identical (no transformation applied).
