# Supplementary material for: A causal inference and Bayesian optimisation framework for modelling multi-trait relationships—Proof-of-concept using Brassica napus seed yield under controlled conditions
Source: PLoS One. 2023 Sep 1;18(9):e0290429. doi: 10.1371/journal.pone.0290429 (PMC10473526; doi:10.1371/journal.pone.0290429)
Supplement: S3 Table — Statistical significance of genotype effect on measured traits estimated by one way ANOVA. Reported p-values were adjusted for multiple hypothesis testing by Benjamini-Hochberg method. Broad sense heritability (H2=σg2/σp2) was estimated from the mean squares components of ANOVA, following [28], using either normalised observed trait values, or model residuals (see methods). Dashes indicate that the trait is not modelled as having any parent traits, and so residual values are the same as for raw values. (DOCX) [file pone.0290429.s010.docx]

| **trait** | **Genotype effect (adjusted p-value)** | | **H^2^** | |
| --- | --- | --- | --- | --- |
|  | **total** | **direct** | **total** | **direct** |
| %PodAbortion M | 2.0E-14 | 2.9E-03 | 0.37 | 0.12 |
| %PodAbortion S | 5.8E-17 | 3.6E-03 | 0.40 | 0.12 |
| BeakLength | 9.7E-26 | 1.9E-07 | 0.74 | 0.39 |
| GynLength | 6.4E-23 | 1.6E-03 | 0.70 | 0.23 |
| Height | 3.0E-46 | - | 0.68 | - |
| NumberFlowers | 6.6E-20 | 2.6E-16 | 0.44 | 0.39 |
| NumberPods M | 8.1E-43 | 9.1E-06 | 0.66 | 0.20 |
| NumberPods S | 3.4E-13 | 5.3E-01 | 0.35 | 0.00 |
| NumberSecondInfl | 7.7E-27 | - | 0.52 | - |
| OilContent | 4.0E-40 | 2.7E-16 | 0.64 | 0.39 |
| OvaryLength | 1.8E-28 | 5.9E-24 | 0.76 | 0.71 |
| OvuleArea | 9.5E-17 | 2.9E-12 | 0.61 | 0.52 |
| OvuleAreaVar | 2.8E-01 | 2.1E-01 | 0.04 | 0.10 |
| OvuleNumber | 5.2E-26 | 3.4E-13 | 0.74 | 0.54 |
| PodLength | 2.8E-33 | 5.5E-17 | 0.59 | 0.40 |
| SeedArea | 3.8E-49 | 4.0E-06 | 0.71 | 0.21 |
| SeedArea M | 3.4E-49 | 2.8E-21 | 0.71 | 0.46 |
| SeedAreaVar | 9.8E-20 | 3.4E-13 | 0.44 | 0.35 |
| SeedCompactness | 5.1E-64 | 5.4E-33 | 0.78 | 0.59 |
| SeedCompactness M | 2.2E-56 | 8.0E-09 | 0.75 | 0.27 |
| SeedNumber | 1.4E-39 | 3.7E-03 | 0.64 | 0.12 |
| SNPP | 4.3E-26 | 2.8E-12 | 0.52 | 0.33 |
| SeedWeight M | 2.6E-22 | 1.8E-06 | 0.47 | 0.21 |
| SeedYield | 2.7E-36 | 6.6E-01 | 0.61 | -0.02 |
| StyleLength | 1.7E-20 | 2.1E-10 | 0.67 | 0.75 |
| TGW | 9.0E-45 | 4.0E-03 | 0.68 | 0.12 |
| TimeToFlower | 1.3E-48 | 1.4E-24 | 0.70 | 0.50 |

**Supplemental Table 3:** Estimated genetic control of phenotypic traits. Statistical significance of genotype effect on measured traits estimated by one way ANOVA. Reported p-values were adjusted for multiple hypothesis testing by Benjamini-Hochberg method. Broad sense heritability ($H^{2}={\sigma_{g}^{2}}/{\sigma_{p}^{2}}$) was estimated from the mean squares components of ANOVA, following (Singh et al., 1993)**,** using either normalised observed trait values, or model residuals (see methods). Dashes indicate that the trait is not modelled as having any parent traits, and so residual values are the same as for raw values.
