# Supplementary material for: A causal inference and Bayesian optimisation framework for modelling multi-trait relationships—Proof-of-concept using Brassica napus seed yield under controlled conditions
Source: PLoS One. 2023 Sep 1;18(9):e0290429. doi: 10.1371/journal.pone.0290429 (PMC10473526; doi:10.1371/journal.pone.0290429)
Supplement: S3 File — Heritability estimation and causal SNP identification. (DOCX) [file pone.0290429.s013.docx]

**Supplemental Methods:**

**Heritability estimation**

In the absence of environmental variation, observed trait values can be expressed as

$$y_{ij}=\mu+g_{i}+ \epsilon_{ij}$$

where the observed value ($y$) of plant $j$ in genotype $i$ is a combination of its mean value ($\mu$), and genetic effect ($g_{i}$) and a stochastic noise effect ($\epsilon_{ij})$. Total broad sense heritability ($H^{2}$) was calculated as ${\sigma_{g}^{2}}/{{(\sigma}_{g}^{2}+\sigma_{\epsilon}^{2})}$ following (Singh et al., 1993) to estimate variances.

When considering causal trait-trait relationships, observed trait values can alternatively be expressed as a function of the values of its parent traits, plus direct genetic and stochastic effects

$y_{ij}=f\left( y_{ij}^{p} \right)+g_{i}^{d}+ \epsilon_{ij}^{d}$.

Child trait values given parent traits were estimated as described above. By subtracting the predicted child trait value ($f\left( y_{ij}^{p} \right)$) from the observed child trait value ($y_{ij}$), the variance in direct genetic $(g_{i}^{d}$) and direct stochastic ($\epsilon_{ij}^{d}$) components was estimated as for total broad sense heritability. “Direct heritability” was calculated as the variance in $g_{i}^{d}$ as a proportion of the variance in $y_{ij}$.

**Simulation of causal SNP identification**

Simulated data was generated in R, using the generative model as shown in **Supplemental Figure** **6a**.

In each experiment, 100 “plants” were simulated, each with their own set of SNPs. Each plant has five parent SNPs, five child SNPs, and 1000 non-causal SNPs. All SNPs are independent Bernoulli random variables with mean = 0.5. For each plant, the value of the child and parent traits were calculated using equations shown in **Supplemental Figure 6a**. For all plants, $\boldsymbol{\beta=\delta=1}$. Values of $\gamma$ and $\sigma$ were varied between experiments as shown in **Supplemental Figure 6b**. Each simulation experiment was repeated ten times.

For each experiment, a linear model to predict the child trait from the parent trait was fitted. SNPs associated with the child trait were inferred by testing for association to either the child trait directly (direct observation) or to the residuals of this model (model residual).

Association of each SNP with was evaluated as the significance of the SNP coefficient in a linear regression model, using a Benjamini-Hochberg corrected p-value threshold, with false discovery rate $\leq$ 0.01.
